# A rank-based marker selection method for high throughput scRNA-seq data

**DOI:** 10.1186/s12859-020-03641-z

**Published:** 2020-10-23

**Authors:** Alexander H. S. Vargo, Anna C. Gilbert

**Affiliations:** 1 Department of Mathematics, University of Michigan, 530 Church Street, Ann Arbor, 48109 USA; 2 Department of Mathematics, Yale University, 10 Hillhouse Ave, New Haven, 06511 USA

**Keywords:** Single cell RNA-seq, Marker selection, Machine learning, Data analysis, Algorithms, Benchmarking

## Abstract

**Background:**

High throughput microfluidic protocols in single cell RNA sequencing (scRNA-seq) collect mRNA counts from up to one million individual cells in a single experiment; this enables high resolution studies of rare cell types and cell development pathways. Determining small sets of genetic markers that can identify specific cell populations is thus one of the major objectives of computational analysis of mRNA counts data. Many tools have been developed for marker selection on single cell data; most of them, however, are based on complex statistical models and handle the multi-class case in an ad-hoc manner.

**Results:**

We introduce RankCorr, a fast method with strong mathematical underpinnings that performs multi-class marker selection in an informed manner. RankCorr proceeds by ranking the mRNA counts data before linearly separating the ranked data using a small number of genes. The step of ranking is intuitively natural for scRNA-seq data and provides a non-parametric method for analyzing count data. In addition, we present several performance measures for evaluating the quality of a set of markers when there is no known ground truth. Using these metrics, we compare the performance of RankCorr to a variety of other marker selection methods on an assortment of experimental and synthetic data sets that range in size from several thousand to one million cells.

**Conclusions:**

According to the metrics introduced in this work, RankCorr is consistently one of most optimal marker selection methods on scRNA-seq data. Most methods show similar overall performance, however; thus, the speed of the algorithm is the most important consideration for large data sets (and comparing the markers selected by several methods can be fruitful). RankCorr is fast enough to easily handle the largest data sets and, as such, it is a useful tool to add into computational pipelines when dealing with high throughput scRNA-seq data. RankCorr software is available for download at https://github.com/ahsv/RankCorrwith extensive documentation.

## Background

In recent years, single cell RNA sequencing (scRNA-seq) has made it possible to characterize cellular diversity by determining detailed gene expression profiles of specific cell types and states ([1, 2]). Furthermore, mRNA data can now be collected from more than one million cells in one experiment due to the development of high throughput microfluidic sequencing protocols [3]. The incorporation of unique molecular identifier (UMI) technology additionally makes it possible to process these raw sequencing data into integer valued read counts (instead of the “counts per million fragments” types of rates that were used in bulk sequencing [1]). Thus, modern scRNA-seq experiments produce massive amounts of integer valued counts data.

These scRNA-seq data exhibit high variance and are sparse (often, approximately 90% of the reads are 0 [4]) for both biological (e.g. transcriptional bursting) and technical (e.g. 3’ bias in UMI based sequencing protocols) reasons. Those characteristics, in combination with the integer valued quality of the counts and the high dimensionality of the data (often, 20,000 genes show nonzero expression levels in an experiment), are such that scRNA-seq data do not match many of the models that underlie common data analysis techniques. For this reason, many specialized tools have been developed to answer biological questions with scRNA-seq data.

One such biological question that has generated a significant amount of study in the scRNA-seq literature is the problem of finding *marker genes*. From a biological perspective, we loosely define marker genes as genes that can be used to identify a given group of cells and distinguish those cells from all other cells or from other specific groups of cells. Usually, these are genes that show higher (or lower) levels of expression in the group of interest compared to the rest of the cell population; this provides simple ways to visualize the cell types and to test for the given cell types in experiments. In practice, certain genes are more desirable markers than others; for example, marker genes that encode surface proteins allow for the physical isolation of cell types via fluorescence-activated cell sorting (FACS).

A multitude of tools for finding marker genes are present in the (sc)RNA-seq literature. These tools often inherently define marker genes to be genes that are *differentially expressed* between two cell populations. That is, in order to find the genes that are useful for separating two populations of cells, a statistical test is applied to each gene in the data set to determine if the distributions of gene expression are different between the two populations: the genes with the most significance are selected as marker genes. The commonly-used analysis tools scanpy [5] and Seurat [6] implement differential expression methods as their default marker selection techniques; see also [7] for a survey of differential expression methods.

Marker selection has also received extensive study in the computer science literature, where it is known as *feature selection*. Given a data set, the goal of feature selection is to determine a (small) subset of the variables (genes) in that data set that are the most “relevant.” In this case, the relevance of a set of variables is defined by some external evaluation function - different feature selection algorithms use a variety of approaches to optimize different relevance functions.

There are generally two main classes of feature selection algorithms: greedy algorithms that select features one-by-one, computing a score at each step to determine the next marker to select (for example, forward- or backward-stepwise selection, see Section 3.3 of [8]; mutual information based methods, see e.g. [9]; and other greedy methods e.g. [10]), and slower algorithms that are based on solving some regularized convex optimization problem (for example LASSO [11], Elastic Nets [12], and other related methods [13]).

A major drawback of many existing feature selection and differential expression algorithms is that they are not designed to handle data that contain more than two cell types. Using a differential expression method, for example, one strategy is to pick a fixed number (e.g., 10) of the statistically most significant genes for each cell type; there may be overlap in the genes selected for different cell types. This strategy does not take into account the fact that some cell types are more difficult to characterize than others, however: one cell type may require more than 10 markers to separate from the other cells, while a different cell type may be separated with only one marker. Setting a significance threshold for the statistical test does not solve this problem; a cell type that is easy to separate from other cells will often exhibit several high significance markers, while a cell type that is difficult to separate might not exhibit any high significance markers.

In this work, we introduce RANKCORR, a feature selection algorithm that addresses the problem of multi-class marker selection on massive data sets in a novel manner^1^. RANKCORR is motivated by the algorithm introduced in [14]; here, we present a fast method for solving the optimization problem from [15] that is at the core of the algorithm from [14]. As a result, RANKCORR runs quickly: it uses computational resources commensurate with several fast and light simple statistical techniques and it can run on data sets that contain over one million cells. In addition, a key step of the RANKCORR method is ranking the scRNA-seq data: this provides a non-parametric way of considering the counts and eliminates the need to normalize the data. Moreover, RANKCORR is a one-vs-all method that selects markers for each cluster based on one input parameter. Instead of providing a score for every gene in each cluster and requiring for the user to manually trim these lists down, RANKCORR selects an informative number of markers for each cluster. In the general case, different numbers of markers will be selected for different clusters. The union of the markers selected for all clusters provides a set of markers that is informative about the entire clustering. Unlike the method of choosing a significance threshold with a differential expression method, cell types that are more difficult to separate from others will generally contribute more markers to the final set. Thus, RANKCORR represents a step towards principled multi-class marker selection when compared to the procedures that are common in most existing methods.

We test the performance of RANKCORR when it is applied to a collection of four experimental UMI counts data sets and an ensemble of synthetic data sets. These data sets contain up to one million cells and include examples of well-differentiated cell types as well as cell differentiation trajectories. Moreover, each data set comes equipped with a cell type classification; we consider classifications that are biologically motivated as well as clusters that are algorithmically created. We refer to the original data source references for their detailed descriptions of clustering and labeling procedures. See Table 1 for a summary of the data sets; full descriptions of the experimental data sets and synthetic data sets can be found in the Methods.

**Table 1:** Data sets considered in this work. The ’genes’ column lists the number of non-zero genes detected in the data set. The peripheral blood mononuclear cell (PBMC) data set from [2] appears twice in this table: ZHENGFILT contains a subset of the full data set ZHENGFULL. See experimental data for more information. ZHENGSIM is a collection of simulated data sets created with the Splatter R package; see the generating synthetic data in the Methods sections for more information

Data set	Cells	Genes	Description	Ground truth clusters	Ref.
Zeisel	3005	4999	mouse neurons	9 (well-separated clusters)	[24]
Paul	2730	3451	mouse myeloid progenitor cells	19 (differentiation trajectory)	[25]
ZhengFull	68579	20387	human PBMCs	11 (some clusters overlap)	[2]
ZhengFilt		5000			
10 x Mouse	1.3 million	24015	mouse neurons	39 (algorithmically generated)	[3]
ZhengSim	5000	varies	simulated from human CD19+ B cells	2	using [26]

Using these data sets, we compare RANKCORR to a diverse set of feature selection methods. We consider feature selection algorithms from the computer science literature, the statistical tests used by default in the in the Seurat [6] and scanpy [5] packages, and several more complex statistical differential expression methods from the scRNA-seq literature. Refer to Table 2 and the marker selection methods section for a detailed list.

**Table 2:** Differential expression methods tested in this paper. The “Data sets” column lists the data sets that each method is tested on in this work. The top block contains the methods that are presented in this work; implementations of these methods can be found in the repository linked in the data availability disclosure. The second block of methods consists of general statistical tests. The third block consists of methods that were designed specifically for scRNA-seq data. The fourth block consists of standard machine learning methods; Log. Reg. stands for logistic regression. We also consider selecting markers randomly without replacement. See the marker selection methods description in the Methods for more information

Method	Data sets	Package	Version	Ref
RankCorr	All	custom		
Spa	Zeisel, Paul	implementation		[14]
t-test	All	scanpy	1.3.7; see text	
Wilcoxon	All		1.3.7; see text	
edgeR	Zeisel, Paul, ZhengFilt	edgeR, rpy2 v2.9.4	3.24.1	[27]
MAST	Zeisel, Paul, ZhengFilt	MAST, rpy2 v2.9.4	1.8.1	[28]
scVI	Zeisel, Paul	Source from GitHub	0.2.4	[29]
Elastic Nets	Zeisel, Paul	scikit-learn [30]	0.20.0	[12]
Log. Reg.	All	scanpy	1.3.7; see text	[31]
Random selection	Zeisel, Paul, ZhengFilt,			
	ZhengFull			

There is currently no definitive ground truth set of markers for any experimental scRNA-seq data set. Known markers for cell types have usually been determined from bulk samples, and treating these as ground truth markers neglects the individual cell resolution of single cell sequencing. Moreover, we argue that the set of known markers is incomplete and that other genes could be used as effectively as (or more effectively than) known markers for many cell types. Indeed, finding new, better markers for rare cell types is one of the coveted promises of single cell sequencing.

Since one goal of marker selection is to discover heretofore unknown markers, we cannot easily evaluate the efficacy of a marker selection algorithm by testing to see if the algorithm recovers a set of previously known markers on experimental scRNA-seq data sets. For this reason we evaluate the quality of the selected markers by measuring how much information the selected markers provide about the given clustering. In this work, we propose several metrics that attempt to quantify this idea.

According to these evaluation metrics, all of the algorithms considered in this manuscript produce reasonable markers, in the sense that they all perform significantly better than choosing genes uniformly at random. In addition, RANKCORR tends to perform well in comparison to the other methods, especially when selecting small numbers of markers. That said, there are generally only small differences between the different marker selection algorithms, and the “best” marker selection method depends on the data set being examined and the evaluation metric in question. It is thus impossible to conclude that any method *always* selects better markers than any of the others.

The major factors that differentiate the methods examined in this work are the computational resources (both physical and temporal) that the methods require. Since the algorithms show similar overall quality, researchers should prefer marker selection methods that are fast and light. This suggests that fast marker selection methods should be preferred over high complexity algorithms. RANKCORR is one of the fastest and lightest algorithms considered in this text, competitive with simple statistical tests. Thus, as a fast and efficient marker selection method that takes a further step towards understanding the multi-class case, RANKCORR is a useful tool to add into computational toolboxes.

### Related work: towards a precise definition of marker genes

In the preceding discussion, we implicitly defined three types of “marker genes”:

- biological markers, i.e. genes whose expression can be used in a laboratory setting to distinguish the cells in one population from the other cells (or from other cell subpopulations);
- genes that are differentially expressed between one cell population and the other cells (or another cell subpopulation);
- and genes that are chosen according to a feature selection algorithm that statistically/mathematically characterizes the relevance of genes to the cell populations in some way (e.g. by minimizing a loss function).

Although we will use these ideas fairly interchangeably throughout this manuscript (referring, for example, to “the markers selected by the algorithm”), it is important to keep in mind the differences between them. For instance, a differentially expressed gene that shows low expression is not a particularly useful biological marker. Indeed, it would be difficult to use a low expression gene to visualize the differences between cell types and inefficient to purify cell populations based on a low expression gene with a FACS sorter.

Several recent marker selection tools start to bridge the gap between differentially expressed genes and biological markers. For example, [16] incorporates a high expression requirement in a heuristic mathematical definition of marker genes, and [17] utilizes a test for differential expression that is robust to small differences between population means. For a given cell type and candidate marker gene, the test used in [17] also incorporates both a lower bound on the number of cells that must express the candidate marker within the cell type and an upper bound on the number of cells that can express a marker outside of the cell type. See the discussion of marker selection methods in the Methods for some further information.

In any case, it is worthwhile to establish a more precise biological definition of a marker gene in order to provide a solid theoretical framework for marker selection. For example, one biological definition of markers requires the cells to be grouped before markers can be determined; this assumes that markers are inherently associated with known cell types or states. This approach is influenced by the computational pipeline that many researchers are currently following (clustering followed by marker selection, see e.g. [18, 19]) and is the approach we consider in this manuscript. An alternative is to define markers as genes that naturally separate the cells into groups in some nice way; the discovered groups would then be classified into different cell types (i.e. allow marker selection to guide clustering). Another recent method [20] defines markers in terms of their overall importance to a clustering, eschewing the notion of markers for specific cell types. Their framework also incorporates finding markers for hierarchical cell type classifications (instead of “flat” clustering). We leave full considerations of rigorous definitions for future work.

### Notation and definitions

Let ***R*** denote the set of real numbers, ***Z*** denote the set of integers, and ***N*** denote the set of natural numbers.

Consider an scRNA-seq experiment that collects gene expression information from *n* cells, and assume that *p* different mRNAs are detected during the experiment. After processing, for each cell that is sequenced, a vector *x*∈***R***^*p*^ is obtained: *x*_*j*_ represents the number of copies of a specific mRNA that was observed during the sequencing procedure. When all *n* cells are sequenced, this results in *n* vectors in ***R***^*p*^, which we arrange into a data matrix *X*∈***R***^*n*×*p*^. The entry *X*_*i*,*j*_ represents the number of counts of gene *j* in cell *i*. Note that this is the *transpose* of the data matrix that is common in the scRNA-seq literature.

Let [ *n*]={1,…,*n*}. For a matrix *X*, let *X*_*i*_ denote column *i* of *X*. Given a vector *x*, let $\mu (x) = \bar {x}$ denote the average of the elements of *x* and let *σ*(*x*) represent the standard deviation of the elements in *x*; that is, $\sigma (x) = \sqrt {\tfrac 1n {\sum \nolimits }_{i=1}^{n} (x_{i} - \mu (x))^{2}}.$ We use the notation ∥*x*∥_*p*_ to represent the *p*-norm of the vector *x*. For example, ∥*x*∥_2_ is the standard Euclidean norm of *x* and $\left \|x\right \|_{1} = {\sum \nolimits }_{i=1}^{n} |x_{i}|$. The notation ∥*x*∥_0_ represents the number of nonzero elements in *x*.

### Ranking scRNA-seq data

The first step of RANKCORR is to *rank* the entries of an scRNA-seq counts matrix *X*. In this section, we make the notion of ranking precise, and we establish some intuition as to why the rank transformation produces intelligible results on scRNA-seq UMI counts data. We can do much more formal analysis in regards to the behavior of the rank transformation on scRNA-seq data; this analysis will appear in an upcoming work.

Consider a vector *x*∈***R***^*n*^. For a given index *i* with 1≤*i*≤*n*, let *S*_*i*_(*x*)={*ℓ*∈[ *n*]:*x*_*ℓ*_<*x*_*i*_} and *E*_*i*_(*x*)={*ℓ*∈[ *n*]:*x*_*ℓ*_=*x*_*i*_} (note that *i*∈*E*_*i*_(*x*)). We have that |*S*_*i*_(*x*)| is the number of elements of *x* that are strictly smaller than *x*_*i*_ and |*E*_*i*_(*x*)| is the number of elements of *x* that are equal to *x*_*i*_ (including *x*_*i*_ itself).

#### **Definition 1**

The *rank transformation* *Φ*:***R***^*n*^→***R***^*n*^ is defined by

1 $$ \Phi(x)_{i} = \left|S_{i}(x)\right| + \frac{\left|E_{i}(x)\right| + 1}{2}.  $$

Note that *Φ*(*x*)_*i*_ is the index of *x*_*i*_ in an ordered version of *x* (i.e. it is the *rank* of *x*_*i*_ in *x*). If multiple elements in *x* are equal, we assign their ranks to be the average of the ranks that would be assigned to those elements (that is, for fixed *i*, all elements *x*_*j*_ for *j*∈*E*_*i*_(*x*) will be assigned the same rank).

#### **Example 1**

Let *n*=5, and consider the point *x*=(17,17,4,308,17). Then *Φ*(*x*)=(3,3,1,5,3). This value will be the same as the rank transformation applied to any point in *x*∈***R***^5^ with *x*_3_<*x*_1_=*x*_2_=*x*_5_<*x*_4_.

Ranking is commonly used in non-parametric statistical tests - ranking scRNA-seq data allows for statistical tests to be performed on the data without specific assumptions about the underlying distribution for the counts. This is important, since models for the counts distribution are continually evolving. As the measurement technology develops, different statistical models become more (or less) appropriate.

**Fig. 1:**
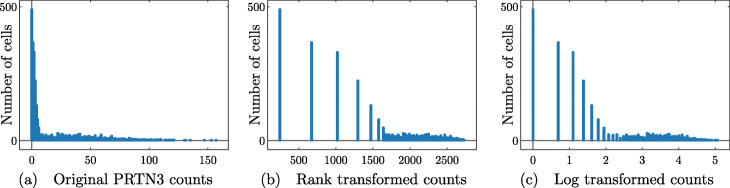
Counts of gene PRTN3 in bone marrow cells in the PAUL data set (See the Methods). Each point corresponds to a cell; the horizontal axis shows the number of reads and the vertical axis shows the number of cells with a fixed number of reads. No library size or cell size normalization has been carried out in these pictures. Note that the tail of the log transformed data is subjectively longer, while the gap between zero counts and nonzero counts appears larger in the rank transformed data

In addition, the rank transformation seems to be especially suited to UMI counts data, which is sparse and has a high dynamic range. When analyzing the expression of a fixed gene *g* across a population of cells, it is intuitive to separate the cells that are observed to express *g* from the cells that are not. Among the cells that express *g*, it is important to distinguish between low expression of *g* and high expression of *g*. The actual counts of *g* in cells with high expression (say a count of 500 vs a count of 1000) are often not especially important. Under the rank transformation, the largest count will be brought adjacent to the second-largest - no gap will be preserved. On the other hand, since there are many entries that are 0, the gap between no expression (a count of 0) and some expression will be significantly expanded (in Eq. (1), the set *E*_*i*_(*x*) will be large for any *i* such that *x*_*i*_=0). See Fig. 1 for a visualization of these ideas on experimental scRNA-seq data.

Stratifying gene expression in this way is intuitively useful for determining the genes that are important in identifying cell types: a gene that shows expression in many cells of a given cell type can be used to separate that cell type from all of the others and thus is a useful marker gene. Thus, by enforcing a large separation between expression and no expression (when compared to the separation between low expression and high expression), it will be easier to identify markers. For these reasons, and since the rank transformation has shown promise in other scRNA-seq tools (for example NODES [21]) we use the rank transform in the RANKCORR marker selection algorithm.

A final note is that a connection can be made between the rank transformation and the log normalization that is commonly performed in the scRNA-seq literature. Often, the counts matrix *X* is normalized by taking *X*_*ij*_↦ log(*X*_*ij*_+1). This is a nonlinear transformation that helps to reduce the gaps between the largest entries of *X* while leaving the entries that were originally 0 unchanged (and preserving much of the gap between “no expression” and “some expression”). With this in mind, the rank transformation can be viewed as a more aggressive log transformation.

## Results

Our results are of two types: (i) algorithmic performance guarantees for RANKCORR and (ii) empirical performance of RANKCORR and its comparison algorithms on scRNA-seq data sets (both benchmark and simulated).

### RANKCORR: algorithmic performance guarantees

RANKCORR is based on the ideas presented in [14]. It is a fast algorithm that chooses an informative number of genes for each cluster by first ranking the scRNA-seq data, and then splitting the clusters in the ranked data with sparse separating hyperplanes that pass through the origin.

A full description of the RankCorr algorithm is found in the Methods. We provide an outline here so as to explain why it is such an efficient algorithm as compared to alternatives. RANKCORR requires three inputs:

- An scRNA-seq counts matrix *X*∈***R***^*n*×*p*^ (*n* cells, *p* genes). The rank transformation provides a non-parametric normalization of the counts data, and thus there is no need to normalize the counts data before starting marker selection.
- A vector of labels *y*∈***Z***^*n*^ that defines a grouping of the cells (*y*_*i*_=*k* means that cell *i* belongs to group *k*). We think of *y* as separating the cells into distinct cell types or cell states, but in general the groups defined by *y* could consist of any arbitrary (non-overlapping) subsets of cells.
- A parameter *s* that indirectly controls the number of markers to select. For a fixed input *X*, increasing *s* will produce more markers.

Before selecting markers, RANKCORR ranks and standardizes the input data *X* to create the matrix $\overline {X}$ defined by

2 $$ \overline{X}_{j} = \frac{\Phi\left(X_{j}\right) - \mu\left(\Phi\left(X_{j}\right)\right)}{\sigma\left(\Phi\left(X_{j}\right)\right)}  $$

where *Φ* is the rank transformation, as defined in (1), and *X*_*j*_ denotes the *j*-th column of *X*^2^. Markers are then selected for the clustering defined by *y* in a one-vs-all manner. For a single group *k*∈*y*, define *τ*∈{±1}^*n*^ such that *τ*_*i*_=+1 if *y*_*i*_=*k* (that is, if cell *i* is in group *k*) and *τ*_*i*_=−1 otherwise; we refer to *τ* as the *cluster indicator vector* for the group *k*. To select markers for group *k*, RANKCORR constructs the vector $\overline {\tau } = \Phi (\tau) - \mu \left (\Phi \left (\tau \right)\right)$. Following this, using the input parameter *s*, the algorithm determines the vector $\hat {\omega }$ as the solution to the optimization problem (3):

3 $$ \begin{aligned} \hat{\omega} = \underset{\omega}{\arg\max} & \sum\limits_{i=1}^{n} \overline{\tau}_{i} \left\langle \overline{x}_{i}, \omega \right\rangle\\ \mathrm{subject\ to\ } & \left\|\omega\right\|_{2} \leq 1, \left\|\omega\right\|_{1} \leq \sqrt{s} \end{aligned}  $$

where $\overline {x}_{i}$ denotes the *i*-th row of $\overline {X}$. RANKCORR returns the genes that have nonzero support in $\hat {\omega }$ as the markers for group *k*.

Note that the output $\hat {\omega }$ to (3) can be viewed as the normal vector to a hyperplane that passes through the origin and attempts to split the cells in group *k* from the cells that aren’t in group *k*. For example, if cell *i* is in group *k* (i.e. *y*_*i*_=*k*), then the term $\overline {\tau }_{i} \langle \overline {x}_{i}, \omega \rangle $ is positive exactly when $\langle \overline {x}_{i}, \omega \rangle >0$. Thus, the objective function in (3) increases when more cells from group *k* are on the same side of the hyperplane with normal vector $\hat {\omega }$.

The optimization (3) was originally introduced in [15] in the context of sparse signal recovery and was adapted to the context of feature selection in a biological setting in [14]. The speed of RANKCORR is due to a fast algorithm (presented in the Methods) that allows us to quickly jump to the solution of the optimization (3) without the use of specialized optimization software.

#### RANKCORR handles multi-class marker selection in a non-trivial one-vs-all manner

To extend RANKCORR to the multi-class scenario, we select markers for each group of cells defined by *y*. In particular, the value of the parameter *s* is fixed and the optimization (3) is run for each group of cells using the fixed value of *s*. To obtain a collection of markers for the clustering defined by *y* as a whole, RANKCORR returns the union of the selected markers from all of the groups. Alternatively, the markers can be kept separate to provide genes that can identify the individual groups.

The effect of fixing *s* across groups is complex. In the full description of RankCorr, we show that, for a fixed cluster with cluster indicator vector *τ*, the markers that RANKCORR selects are the genes that have the highest (in magnitude) Spearman correlation with *τ*. That is, similar to a differential expression method, RANKCORR can be thought of as generating lists of gene scores (one for each group) that are used to select the proper markers - instead of *p*-values, the scores considered by RANKCORR are magnitudes of specific Spearman correlations.

The parameter *s* does not directly control the number of high-correlation markers that are selected, however, and fixing *s* results in different numbers of markers for each cluster. Thus, the set of markers selected by RANKCORR is different from the set obtained by choosing a constant number of top scoring genes for each cluster. Additionally, fixing *s* is not equivalent to picking a correlation threshold *ρ* and selecting all genes that exhibit a Spearman correlation greater than *ρ* with any cluster indicator vector. Determining a precise characterization of the numbers of markers selected for each cluster is left for future work.

A key result is that RANKCORR contains a new method of merging lists of scores that is based on an algorithm with known performance guarantees [15]. Whether or not this one-vs-all method is an improvement over common heuristics for merging lists requires further exploration. There is some evidence, collected using simple synthetic test data, that selecting a constant number genes with the top Spearman correlation scores for each cluster results in comparable performance to RANKCORR. This has not been studied in the context of experimental data, however, and does not lead to any appreciable time savings over RANKCORR. It is also possible that the merging method used by RANKCORR could be adapted to work with *p*-values and provide an alternative method of merging the lists that are produced by differential expression methods. Thus we focus on RANKCORR as it is currently presented.

### Empirical performance of RANKCORR

In the remainder of this Results, we present evidence that RANKCORR selects markers that are generally similar in quality to (or better than) the markers that are selected by other commonly used marker selection methods. Moreover, RANKCORR runs quickly, and only requires computational resources comparable to those required to run simple statistical tests. Thus, RANKCORR is a useful marker selection tool for researchers to add to their computational libraries.

We evaluated the performance of RANKCORR on four experimental data sets and a collection of synthetic data sets. These data sets are listed in Table 1; see the experimental data and synthetic data descriptions in the Methods for more information (including further details about the ground truth clusters considered in this analysis). We compare RANKCORR to the marker selection methods listed in the leftmost column of Table 2. The Wilcoxon method is the default marker selection technique in the Seurat [6] package, while the scanpy [5] package defaults to the version of the t-test that we include here. See the marker selection methods section for more implementation details.

#### Evaluation of marker sets when ground truth markers are not known

To interpret, evaluate, and simply to present our results, we must quantify how much information a set of selected markers provides about a given clustering when ground truth markers are not known for certain (e.g., when selecting markers on an experimental data set). We propose two general procedures to accomplish this and present results using these:

- Supervised classification: train a classifier on the data contained in the selected markers using the ground truth clustering as the target output.
- Unsupervised clustering: cluster the cells using the information in the set of selected markers without reference to the ground truth clustering.

In this study, we implemented algorithms that accomplish each general procedure. For each algorithm, we considered several different evaluation metrics. A summary of these algorithms and marker set evaluation metrics is found in Table 3, along with the abbreviations that we will use to refer to the metrics.

**Table 3:** Evaluation metrics for marker sets on experimental data. The “Average precision” metric is a weighted average of precision over the clusters. The Matthews correlation coefficient is a summary statistic that incorporates all information from the confusion matrix. See [22] for more information about the classification metrics and [23] for more information about the clustering metrics

General procedure	Methods	Metrics
Supervised classification	Nearest centroid classifier (NCC)Random forests classifier (RFC)	Classification error (1 - accuracy)
		Average precision
		Matthews correlation coefficient
Unsupervised clustering	Louvain clustering	Adjusted rand index (ARI)
		Adjusted mutual information (AMI)
		Fowlkes-Mallows score (FMS)

The three supervised classification metrics (error rate, precision, and Matthews correlation coefficient) generally provide similar information. Thus, for most selected marker sets, we present results from five of the metrics (NCC classification error, RFC classification error, ARI, AMI, and FMS). The precision and Matthews correlation coefficient data can be found in Additional file 1. See the marker evaluation metrics in the Methods for further details about these metrics.

It is important to note that these metrics represent some summary statistical information about the selected markers - they do not capture the full information contained in a set of genes. Results on synthetic data suggest that the metrics are informative but not fine-grained enough to capture all differences between methods. Therefore, we would advise considering these metrics as “tests” for marker selection methods; that is, these metrics should mostly be used to identify marker selection methods that don’t perform well.

We compute each of the metrics using 5-fold cross-validation in order to reduce overfitting (see Section 7.10 of [8]). The timing information reported in the following sections represents the time needed to select markers on one fold.

#### Evaluating RANKCORR on experimental data

**Fig. 2:**
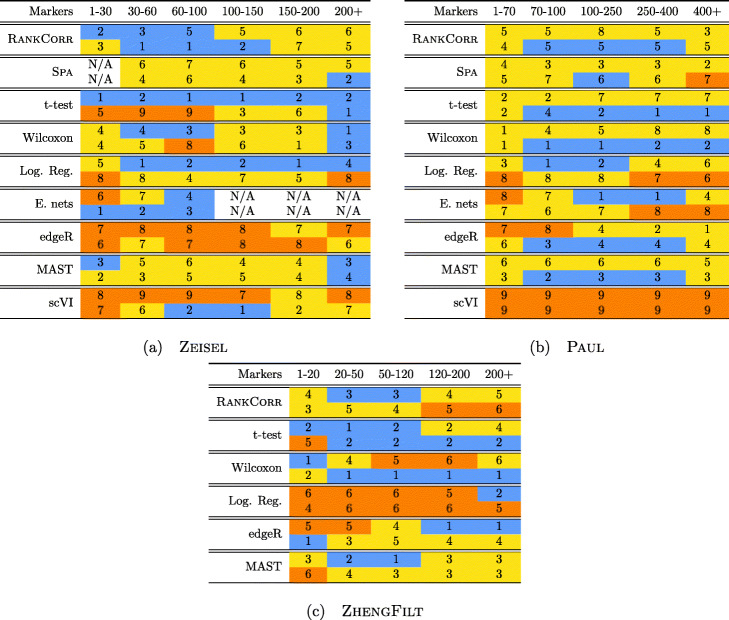
Performance of the marker selection methods on the (**a**) ZEISEL, (**b**) (PAUL), and (**c**) ZHENGFILT data sets as the number of selected markers is varied. There are two rows for each method; the first row for each method represents the classification metrics and the second row represents the clustering metrics. Blue indicates better performance than the other methods; orange indicates notably worse performance than the other methods. The marker bins are chosen to emphasize certain features in Figs. 4, 5, 6, 7, 8, 9 and 10; these figures present the values of the evaluation metrics for the different data sets. The values in the boxes correspond to a ranking of the methods, with 1 being the best method in the marker range. The classification and clustering results are ranked separately. Further notes: (a) All of the methods perform well on the ZEISEL data set - an orange box here does not indicate poor performance, but rather that other methods outperformed the orange one. (b) Many of the methods showed nearly identical performance according to the classification metrics; thus, this table contains many yellow boxes

Performance summaries of the of the methods on the ZEISEL, PAUL, and ZHENGFILT data sets are presented in Fig. 2. In this figure, the colors of the boxes indicate relative performance: a blue box indicates performance that is better than the majority of the other methods, a yellow box indicates median performance, and an orange box indicates performance that is worse than the other methods. The coloring in the figures is based on Figs. 4, 5, 6, 7, 8, 9 and 10; the numbers of markers in the bins (in the top row) are selected to emphasize features found in these plots. The first row for each method represents the classification metrics and the second row represents the clustering metrics.

The values in the columns in Fig. 2 correspond to a heuristic ranking of the methods, with 1 the optimal method (on average) in the indicated range of markers; see the Methods for a full description of how the ranking is calculated. The classification metrics and clustering metrics are ranked separately (so that each column contains two full rankings of the methods; e.g. in Fig. 2b, every column contains the numbers 1 to 9 twice). Since these numbers are ranks, they do not capture the magnitude of the gaps in performance between methods. For example, if two methods differ by one rank (e.g. the method ranked 2 vs the method ranked 3), there could be a large gap in performance between the two methods. The colors of the cells are meant to capture the larger differences between (tiers of) methods, and methods with the same color in a column perform comparably (regardless of the difference in rank). For example, in some cases, the top four methods (ranked 1 through 4) are similar to each other and clearly better than the others, so they will be colored blue. In other cases, no method will appear significantly better than the others, so none of the boxes will be colored blue.

The ZHENGFULL and 10XMOUSE data sets were too large for the majority of the methods to handle; thus, data were only collected for the RANKCORR, t-test, logistic regression, and Wilcoxon methods (the fastest methods) on these data sets. We include the 10XMOUSE data specifically as a stress test to determine the methods that can handle the largest data sets. It is impressive that these methods are able to run on such a large data set in a reasonable amount of time. The performance characteristics of the methods on these data sets are found later in this section.

Overall, the different methods select sets of markers that are of similar quality: the performance of any “optimal” method is usually not much better than several of its competitors. For example, the true differences in performance between the yellow and blue boxes in Fig. 2 are often quite small. In addition, there is no method that consistently selects the best markers. The optimal method depends on the choice of data set, the evaluation metric, and the number of markers that are selected.

That said, the RANKCORR method tends to perform well on these data sets: it is generally competitive with the best methods in terms of performance. In particular, it especially excels when selecting less than 100 markers on all three data sets according to both the clustering and classification metrics.

**Fig. 3:**
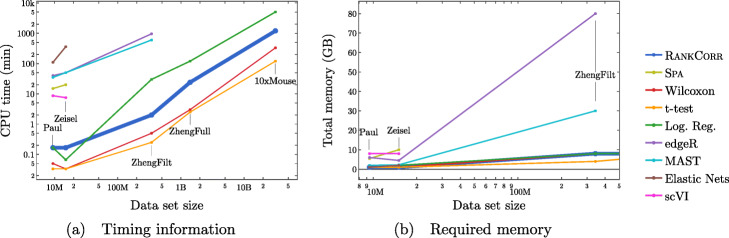
Computational resources used by the marker selection methods. In both figures, the data set size is the number of entries in the data matrix *X*: it is given by *n*×*p*, the number of cells times the number of genes. The data sets that we consider in this work are indicated in the figures. The total computation time required to select markers on one fold (CPU time, calculated as number of processors used multiplied by the time taken for marker selection) is shown in (**a**); the total memory required during these trials is shown in (**b**). Elastic nets scales poorly in (**a**), so it is only run on PAUL and ZEISEL. Both edgeR and MAST are limited by memory on ZHENGFILT (see (**b**)); this prevents their application to the larger data sets. scVI also requires a GPU while it is running; this prevents us from testing it on the larger data sets. RANKCORR, the t-test, Wilcoxon, and logistic regression all use 8 GB to run on ZHENGFULL and 80 GB to run on 10XMOUSE. See the marker selection methods for more details

Since the algorithms generally exhibit similar performances under the metrics considered in this work, efficient algorithms have a significant advantage. The computational resources (total computer time and memory) required to select markers on the experimental data sets are presented in Fig. 3. The fastest and lightest methods are RANKCORR, the t-test, and Wilcoxon: notably, RANKCORR is nearly as fast and light as the two very simple statistical methods (the t-test and Wilcoxon). It is thus these three methods that show a clear advantage over the other methods for working with experimental data. Logistic regression also runs quickly on the smaller data sets but does not scale as well as the three methods mentioned above and significantly slows down on the larger data sets. In addition, logistic regression shows inconsistent performance and is often one of the worst performers when selecting small numbers of markers. The other methods are significantly slower or require large computational resources compared to the size of the data set: see Fig. 3 for further discussion.

**Fig. 4:**
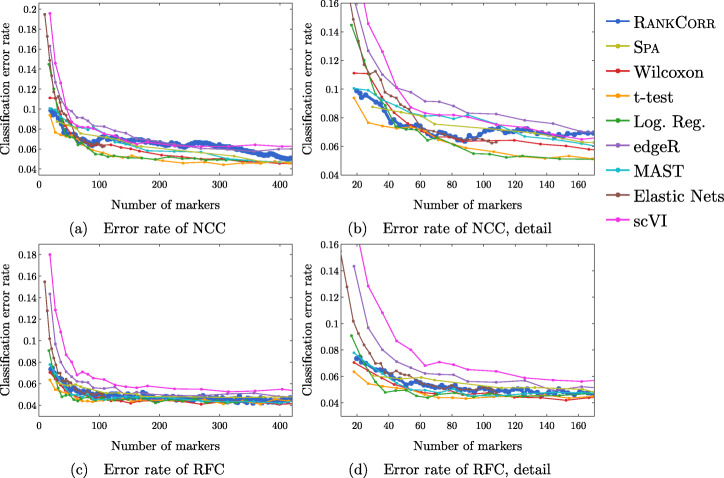
Error rate of both the nearest centroids classifier (NCC; (**a**) and (**b**)) and the random forests classifier (RFC; (**c**) and (**d**)) on the Zeisel data set. Figure (**b**) (respectively (**d**)) is a detailed image of the error rate of the different methods using the NCC (respectively RFC) when smaller numbers of markers are selected

**Fig. 5:**
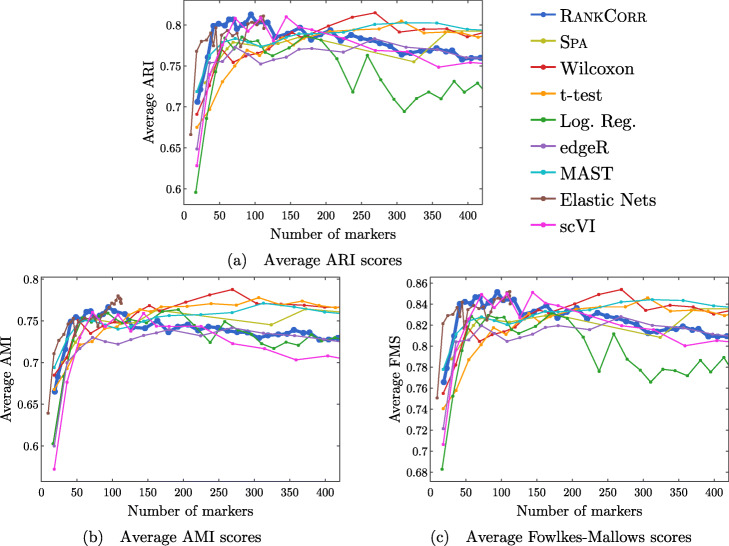
Clustering performance metrics vs total number of markers selected for marker selection methods on the ZEISEL data set. The ARI score is shown in (**a**), the AMI score is shown in (**b**), and the Fowlkes-Mallows score is shown in (**c**). The clustering is carried out using 5-fold cross validation and scores are averaged across folds

**Fig. 6:**
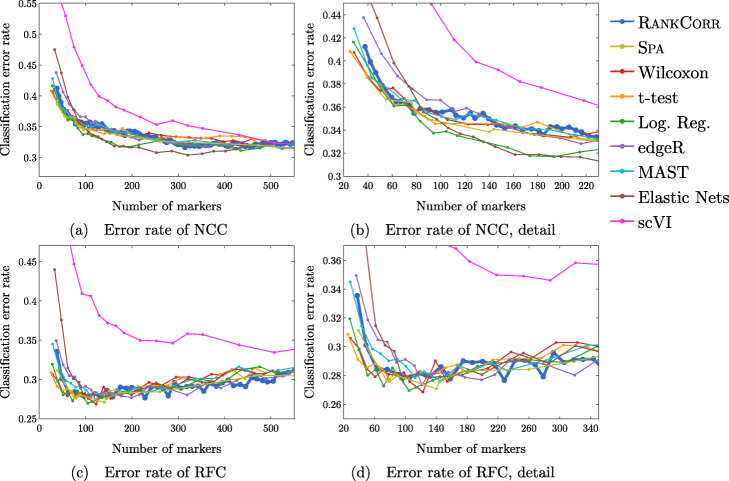
Error rates of both the nearest centroids classifier (NCC; (**a**) and (**b**)) and the random forests classifier (RFC; (**c**) and (**d**)) on the Paul data set. Figure (**b**) (respectively (**d**)) is a detailed image of the error rate of the different methods using the NCC (respectively RFC) when smaller numbers of markers are selected. Figure (**b**) details up to 220 total markers to make clear how similar the methods perform when small numbers of markers are selected. Figure (**d**) examines up to 350 total markers to detail the performance of the methods when small numbers of markers are selected as well as get an idea for the increasing behavior and noisy nature of the curves

**Fig. 7:**
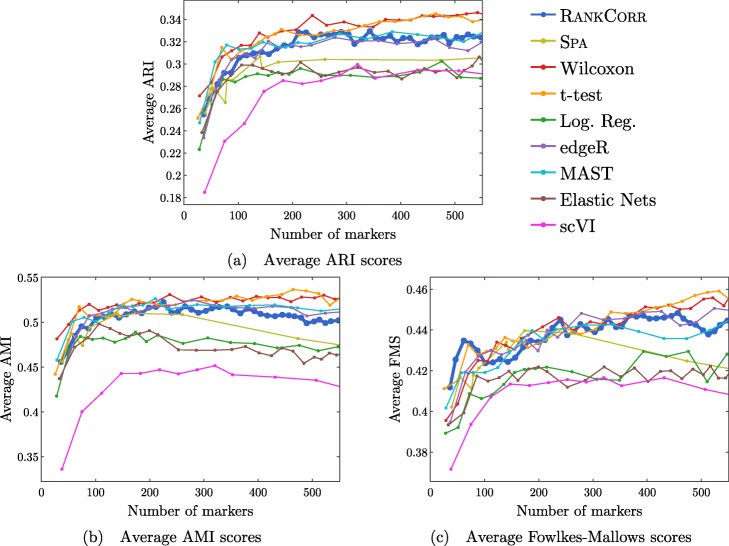
Clustering performance metrics vs total number of markers selected for marker selection methods on the PAUL data set. The ARI score is shown in (**a**), the AMI score is shown in (**b**), and the Fowlkes-Mallows score is shown in (**c**). The clustering is carried out using 5-fold cross validation and scores are averaged across folds

**Fig. 8:**
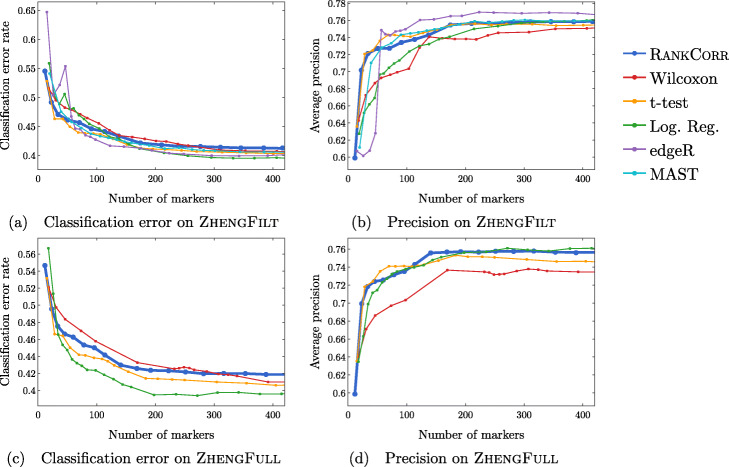
Accuracy and precision of the nearest centroids classifier on the ZHENG data sets using the bulk labels. The top row corresponds to the ZHENGFILT data set and the bottom row corresponds to the ZHENGFULL data set

**Fig. 9:**
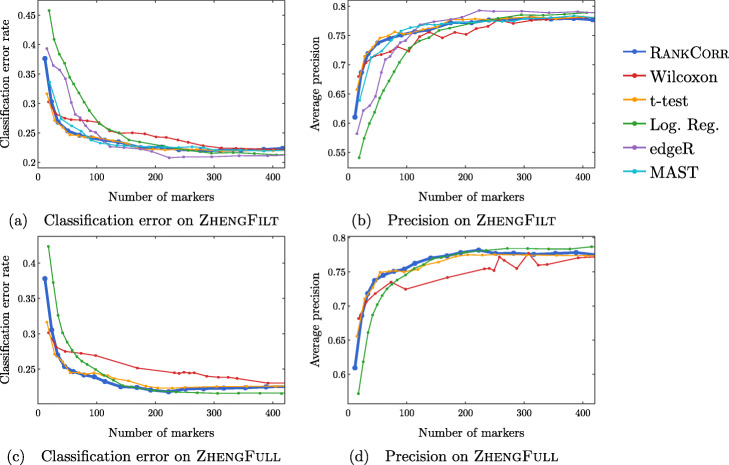
Accuracy and precision of the random forests classifier on the ZHENG data sets using the bulk labels. The top row corresponds to the ZHENGFILT data set and the bottom row corresponds to the ZHENGFULL data set

**Fig. 10:**
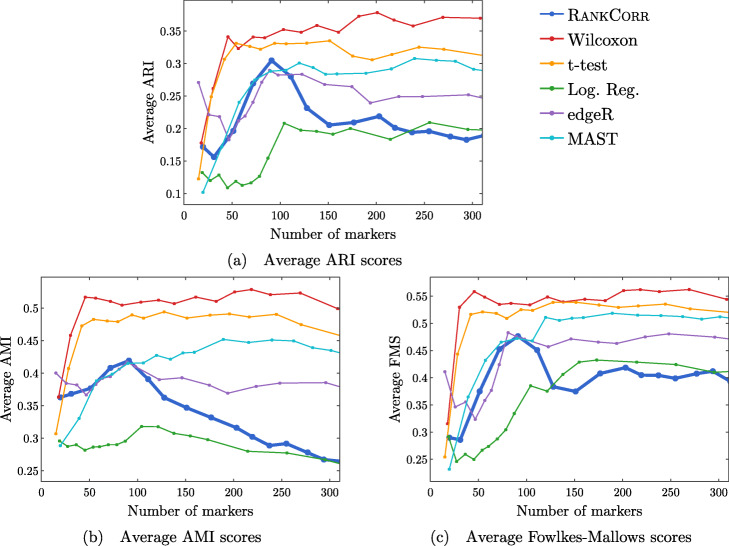
Clustering metrics on the ZHENGFILT data set

These results support the idea that RANKCORR is a worthwhile marker selection method to consider (along side other fast methods) when analyzing massive UMI data sets. In the rest of this section, we give performance results on each specific data set.

#### The marker selection methods perform well on the ZEISEL data set

The classification error rates of the nearest centroid and random forests classifiers on the ZEISEL data set are presented in Fig. 4. The error rates are very low: it requires only 100 markers (an average of 11 markers per cluster) to reach an error rate lower than 5% for most methods using the RFC. The ARI, AMI, and FM scores, reported in Fig. 5, are also high (good) for all methods. Only a small number of markers were selected by elastic nets on the ZEISEL data set; thus, the elastic nets curves end before the others. Figure 2a contains a summary of the data presented in Figs. 4 and 5. The computational resources required by the methods are presented in Fig. 3, where it is clear that the RANKCORR, t-test, Wilcoxon, and logistic regression methods all run quickly on the ZEISEL data set and require few resources in comparison to the other methods.

The ground truth clustering that we consider on the ZEISEL data set is biologically motivated and contains nine clusters that are generally well separated (they represent distinct cell types) [24]. Most of the methods tested here produce markers that provide a significant amount of information about this ground truth clustering; these results thus represent a biological verification of the marker selection methods. That is, in this ideal biological scenario (a data set with highly discrete cell types) the (mathematically or statistically defined) markers that are chosen by the methods are biologically informative and can be used as real (biological) markers.

This suggests that, when selecting markers on a data set that is well clustered, it is useful to examine several marker selection algorithms to get different perspectives on which genes are most important. Marker selection algorithms that can run using only small amounts of resources, such as RANKCORR (see Fig. 3), thus have an advantage over the other methods.

In addition to this, RANKCORR is the only method that shows high performance when selecting fewer than 100 markers in both the clustering and classification metrics. Most researchers will be looking for small numbers of markers for their data sets; thus RANKCORR stands out as a promising method on the ZEISEL data set. Note also that RANKCORR generally outperforms SPA in the clustering metrics and is competitive with SPA in the classification metrics: RANKCORR is both faster than SPA and selects a generally more informative set of markers than SPA on the ZEISEL data set. Therefore, the performance on the ZEISEL data set is evidence for the fact that RANKCORR is a useful adaptation of SPA [14] for sparse UMI counts scRNA-seq data.

Finally, these data illustrate how the different evaluation metrics provide different statistical snapshots into the information contained in a set of markers. For example, logistic regression performs significantly worse than all of the other methods when large numbers of markers are selected according to the ARI and FMS plots. In the supervised classification trials, however, the logistic regression method performs competitively with the other methods. When no information about the ground truth clustering is provided, the performance of logistic regression on the ZEISEL data sets drops considerably. Despite the good results in the supervised clustering plots (that could be due to quickly selecting a small number of useful markers) it is reasonable to conclude that logistic regression selects many uninformative genes (in comparison to the other methods) as more markers are selected. It is best to think of the metrics as tests that can identify the marker selection methods that don’t perform well.

#### Marker selection algorithms struggle with the cell types defined along the cell differentiation trajectory in the PAUL data set

The PAUL data set consists of bone marrow cells and contains 19 clusters [25]. The clusters lie along a cell differentiation trajectory; therefore, it is reasonable that it would be difficult to separate the clusters or to accurately reproduce the clustering into discrete cell types. The Paul data set thus represents an adversarial example for these marker selection algorithms.

Figure 6 shows the performance of marker selection algorithms on the PAUL data set as evaluated by the supervised classification metrics. It is not surprising to see relatively high clustering error rates: the rates are always larger than 30% for the NCC and reach a minimum of around 27% with the RFC. Since there are 19 clusters, this is still much better then classifying the cells at random. The dependence of the ARI, AMI, and FM clustering scores on the number of markers selected is plotted for the different marker selection algorithms in Fig. 7. The values of the scores are all in low to medium ranges for all marker selection algorithms.

All of the scores produced by all of the methods on the PAUL data set are significantly worse than the metrics on the ZEISEL data set; however, the methods perform considerably better than markers selected uniformly at random (see Additional file 1, Figures 5–7 for this comparison). This is sensible, since it should intuitively be difficult to reproduce a discrete clustering that has been assigned along a continuous path. The notion of discrete cell types does not fit well with a cell differentiation trajectory; the poor score levels reflect the necessity to come up with a better mathematical description of a trajectory for the purposes of marker selection.

The ARI values are especially low on the PAUL data set, and the methods consistently produce lower ARI values than AMI values. This is a change from the ZEISEL data set, where the ARI scores were higher than the AMI scores (and the FMSs were the highest of all). The aspects of the data sets that change the relative ordering of the metrics are unclear; it must be the data sets that influence this change, however, since the change persists across the marker selection algorithms. Designing a metric for benchmarking marker selection algorithms is itself a difficult task, and the optimal metric to consider could depend on the data set in question.

A summary of the relative performance of the marker selection algorithms on the Paul data set is presented in Fig. 2b. All of the marker selection methods perform quite similarly on the PAUL data set. The RANKCORR algorithm is one of only three methods that always performs nearly optimally under every metric examined here; the others are the t-test and MAST. In addition, RANKCORR always performs well when selecting small numbers of markers, and shows exceptional performance in this regime under the Fowlkes-Mallows clustering metric. Combined with the facts that RANKCORR is fast to run and requires low computational resources, this shows that RANKCORR is a useful marker selection method to add to computational pipelines.

#### Results on the ZHENGFULL and ZHENGFILT data sets

Here, we examine the data set consisting of 68k peripheral blood mononuclear cells (PBMCs) from [2]; it contains data from more than 30 times the number of cells in either the PAUL or ZEISEL data sets. This is more representative of the sizes of the data sets that we are interested in working with. The ground truth clustering that we consider is the labeling obtained in [2] by correlation with bulk profiles (biologically motivated “bulk labels”). There are 11 cell types in this clustering. See the section describing the data sets (in the Methods) for more information.

The ZHENG data sets contain some distinct clusters (e.g. B cells), as well as some clusters that are highly overlapping (e.g. different types of T cells). There are no specific cell differentiation trajectories (that we are aware of), but the overlapping clusters provide a challenge for the marker selection methods. Thus, we expect to see performance benchmarks between those of PAUL and ZEISEL.

We mostly focus on ZHENGFILT, a version of the data set that is filtered to only include the information from the top 5000 most variable genes. We also consider the performance of the most efficient algorithms (RANKCORR, logistic regression, Wilcoxon, and the t-test) on ZHENGFULL, the data set containing all of the genes, to check for any differences. Extrapolating from Fig. 3, it would be infeasible to run the other methods on ZHENGFULL. Here we also begin to see that logistic regression scales worse than the other methods: it is already becoming slow and computationally heavy on “only” 68 thousand cells.

Figure 8 focuses on the performance of the methods when the NCC is used for classification; corresponding data using the RFC is found in Fig. 9. Unlike the PAUL and ZEISEL data sets, the precision curves are slightly different in some occasions, and thus they are presented here. In particular, the precision of these methods is significantly higher than their accuracy. Neither the classification accuracy nor the precision changes by very much when we filter from the full gene set (Figs. 8c,d and 9c,d) to the 5000 most variable genes (Figs. 8a,b and 9a,b). In general, this filtering very slightly increases both the accuracy and precision of the t-test, Wilcoxon, and RANKCORR methods, while it worsens the performance of the logistic regression method. This suggests that enough marker genes are kept by this variable gene filtering process to maintain accurate marker selection.

Overall, the classification error rates according to the NCC for these data are quite high, and don’t level off (to a minimum value of approximately 40%) until around 200 markers are selected; this corresponds to an average of around 18 unique markers per cluster. For very small numbers of markers selected, the classification error rates obtained from the NCC are quite high (around 55%).

The error rates using the RFC are decreased significantly compared to the NCC, and level off to approximately 22% when large numbers of markers are selected. The error again does not completely level off until around 200 total markers are selected, but there is a steeper initial descent. This steep initial descent in error rates could appear as the large groups of cells are separated from each other (e.g. B cells from T cells) and the slower improvement from 100 to 200 of total markers selected could be the methods fine-tuning the more difficult clusters (e.g. Regulatory T from Helper T). The error rates are between those observed in PAUL and ZEISEL. On the other hand, the error rates for the NCC classifier are much higher than expected.

We focus on the ZHENGFILT data set for the clustering metrics. This is due to the fact that the classification metrics are changed only slightly between ZHENGFILT and ZHENGFULL as well as the fact that Louvain clustering on the large ZHENG data set is itself time and resource intensive. The clustering metrics on the ZHENGFILT data set are presented in Fig. 10. All three scores are generally quite low, though they are again mostly much higher than random marker selection. The performance of random marker selection can be found in Additional file 1, Figure 12.

A summary of the performance of the marker selection algorithms on the ZHENGFILT data set is presented in Fig. 2c. Apart from the t-test, the methods show inconsistent performance when comparing the clustering metrics to the classification methods. For example, the edgeR method exhibits the top performance on the ZHENGFILT data set after more than 50-100 unique markers are selected according to the classification metrics. The classification metrics show edgeR as one of the worst methods when choosing less than 50 unique markers, however. This is in direct contradiction to the clustering metrics, where edgeR is always the best method for the smallest (∼20) total numbers of markers selected, and it then shows performance in the middle of the other methods as larger numbers of markers are selected.

It is possible that changing the number of nearest neighbours considered in the Louvain clustering would produce more consistent data. Although the clustering metrics did not appear to change significantly when altering the number of nearest neighbours on the previous data sets (see the Louvain parameter selection information in the Methods), the ZHENGFILT data set is much larger than those previous data sets; the larger number of cells may necessitate the use of information from more nearest neighbors to recreate the full clustering structure.

It is also possible that the bulk labels that are used for the ground truth are difficult to reproduce through the Louvain algorithm. We generated a clustering that visually looked like the bulk labels via the Louvain algorithm (it appears in Additional file 1, Figure 18); the ARI, AMI, and FMS values for the generated Louvain clustering compared to the bulk labels are in the ranges produced by the Wilcoxon and t-test methods (not larger than the scores here). In addition, the top ARI and AMI scores (produced by the Wilcoxon and the t-test methods) are comparable to (or only slightly better than) the scores on the PAUL data set (Fig. 7). This runs counter to our expectations: the PAUL data set contains a cell differentiation trajectory, with no real clusters that are easy to separate out, while the ZHENG data sets contain several clusters that are well separated. It is possible that the bulk labels produce clusters that are more mixed than it appears in a UMAP plot.

In any case, the disparity between the different types of scores further emphasizes the fact that the classification and clustering metrics provide different ways of looking at the information contained in a selected set of markers. Methods that perform well according to both types of metrics should be preferred.

Following this logic, the t-test produces the overall best results on the ZHENGFILT data set. It performs well under the classification metrics, especially for small numbers of total markers selected. In addition, it is consistently competitive with the best method (Wilcoxon) according to the clustering metrics.

Nonetheless, on the whole, RANKCORR performs approximately as well as the t-test, especially when selecting smaller numbers of markers. In particular, RANKCORR shows nearly optimal performance on the ZHENGFILT data set under the classification metrics. It performs poorly according to the clustering metrics when selecting more than 120 total markers, however, though it is still competitive with logistic regression in this domain. Still, the good performance when selecting less than 120 markers supports the notion that RANKCORR is a useful analytical resource for researchers to consider.

#### Marker selection on the 1 million cell 10XMOUSE data set

We consider the 10XMOUSE data set: it consists of 1.3 million mouse neurons generated using 10x protocols [3]. The “ground truth” clustering that we examine in this case was algorithmically generated without any biological verification or interpretation (see the section about data sets in the Methods). We include this data set as a stress test for the methods and therefore we do not perform any variable gene selection before running the marker selection algorithms (to keep the data set as large as possible). We also only consider the four fastest and lightest methods (RANKCORR, the t-test, Wilcoxon, and logistic regression) as these are the only methods considered in this work that could possibly produce results in a reasonable amount of time on this data set.

The classification error rates of the four methods according to the NCC classifier show behavior similar to the other data sets that we have examined in this manuscript: starting out relatively high when selecting a small number of markers, then rapidly decreasing for a short period until becoming nearly constant as a larger number of markers are selected. See Additional file 1, Figure 13 for the NCC curves.

The error rates of the methods approach approximately 25% as the number of markers selected increases. There are 39 clusters in the “ground truth” clustering that we examine here - thus, the error rates produced by all of the methods are much lower than the error rate expected from random classification. In Additional file 1, Figure 13, we see that the logistic regression method performs the best overall, and that RANKCORR consistently shows the highest error rate. The largest difference between the RANKCORR curve and the logistic regression curve is only around 3%, however. In addition, as mentioned above, logistic regression is the slowest method by far on this data set - extra accuracy is not worth much if the method is not able to finish running.

Because a biologically motivated or interpreted clustering may be quite different from the clustering used here and because the classification error rate does not capture the full information in a set of markers (and thus similar error rates are not necessarily an accurate indication of the relative performance of methods), it is only possible to conclude that all four methods examined here show similar performance on the 10XMOUSE dataset. The RANKCORR method produces useful markers, runs in a competitive amount of time, and takes a step towards selecting a smart set of markers for each cluster (rather than the same number of markers per cluster). It is impressive that these methods are able to run on such a massive data set.

The implementation of the RFC in scikit-learn was quite slow on the large 10XMOUSE data set, and thus we do not compare the methods via the RFC. From the smaller data sets, we might expect that the random forest classifier produces curves that are shaped similarly to the ones in Figure 13 in Additional file 1 but are shifted down to a lower error rate. This is indeed what we see for the RANKCORR method: a comparison of the RFC and the NCC is shown in Additional file 1, Figure 14. Each point on this RFC curve took over 3 hours on 10 processors to generate; the largest point took over 15 hours. The Louvain clustering method was also too slow to compute any clustering error rates for the markers selected here. This is a situation where the marker selection algorithms are faster than almost all of the evaluation metrics (emphasizing the continued need for good marker set evaluation metrics).

### Comparison of marker selection methods on synthetic data

We have evaluated RANKCORR on synthetic data sets that are designed to look like experimental scRNA-seq data. In each synthetic data set that we consider, there is a known ground truth set of markers, and all genes that are not markers are statistically identical across the cell populations. Thus, we can present the actual precision of the marker selection methods as well as ROC curves. Precision is an especially important metric for marker selection - it is desirable for an algorithm to select genes that truly separate the two data sets (rather than genes that are statistically identical across the two populations). The values of precision, TPR, and FPR are computed without cross-validation, since the entire set (of genes) in each data set is test data - there is no training to be done. We additionally examine the classification error metric that was introduced in Table 3. We still use 5-fold cross-validation to compute this metric.

Since the speed of a marker selection algorithm has been observed as an important factor for use on experimental data, we compare RANKCORR only to the fastest methods: the t-test, Wilcoxon, and logistic regression.

**Fig. 11:**
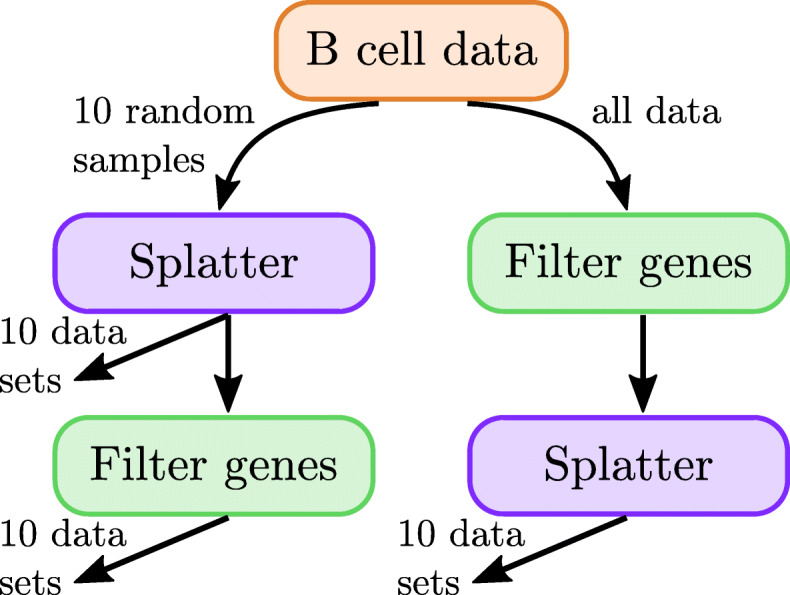
Set up of the simulated data. We consider 3 conditions: all genes used for simulation, filtering after simulation, and filtering before simulation. On the left side of this diagramme, we produce 10 data sets by using all genes in simulation, and 10 more by filtering down to the 5000 most variable genes after simulation. These “filtering after simulation” data sets contain a subset of the information from the “all genes used for simulation” data sets. On the right hand side, we produce 10 data sets by filtering down to the 5000 most variable before simulation

See the synthetic data generation subsection in the Methods for a full description of the data generation process. See also Fig. 11 for an outline of the design. In short, we generate 20 different synthetic data sets; each simulated data set consists of 5000 cells that are split into two groups, and 10% of the genes are differentially expressed between these groups.

For the purposes of computational efficiency, many data analysis pipelines reduce input data to a subset of the most variable genes before selecting markers. Thus, we examine synthetic data sets that are filtered down to the 5000 most variable genes in addition to unfiltered data sets. In 10 samples, we filter before simulating (and simulate 5000 genes); in the other 10 samples, we simulate without filtering (and simulate as many nonzero genes as there were in the input data, usually around 12000 genes). From each data set that was simulated without filtering, we produce another data set by filtering down to the 5000 most variable genes. This results in three simulation conditions (all genes used for simulation, filtering before simulation, and filtering after simulation) and a total of 30 data sets. See Fig. 11.

Apart from the t-test data, each curve presented in this section represents the average across all 10 simulated data sets that are relevant to the curve. For the t-test, one of the trials in each simulation condition produced genes with tied *p*-values. This resulted in situations where it was impossible to select the top *k* genes in a stable manner; thus, these data sets were ignored and the t-test precision, TPR, and FPR curves each represent the average of the 9 data sets that are relevant to the curve.

The differentially expressed genes are chosen randomly; thus many of them show low expression levels (often expressed in less than 10 cells) and are difficult to detect. In general, marker selection methods should not select genes with very low expression levels (since these genes are not particularly useful as markers when all cell types have large enough populations). Thus, we do not present information about the recall here.

#### Simulated data illuminates the precise performance characteristics of marker selection methods

**Fig. 12:**
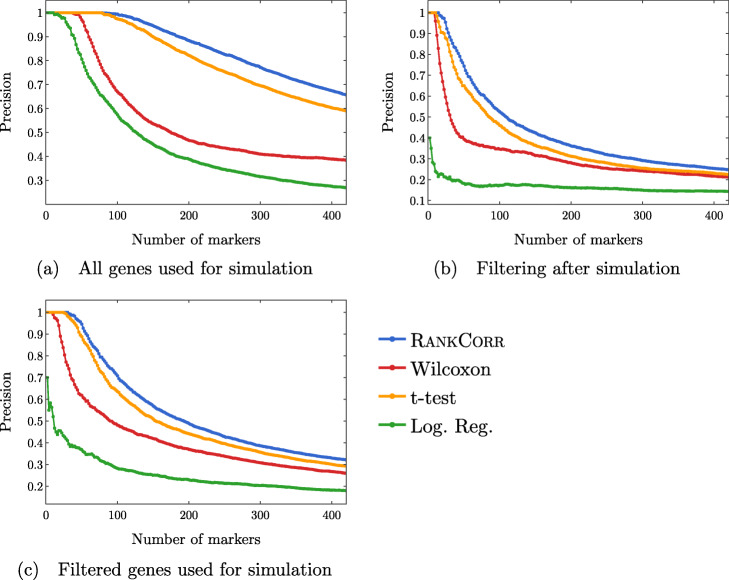
Precision of the marker selection methods versus the number of markers selected for the first 400 markers selected. Each sub-figure corresponds to a simulation method and the four lines correspond to the different marker selection algorithms. The RANKCORR method consistently shows the highest precision across all three simulation methods

In Fig. 12, we examine the precision of the marker selection algorithms for the first 400 unique genes selected. It is promising to see that RANKCORR produces the highest precision in marker selection across all of the simulation methods. The t-test is second, the Wilcoxon method is third, and logistic regression consistently exhibits the lowest precision.

Examining Fig. 12 more closely, we see that the methods generally start off with high precision that decreases as more markers are selected (each data set contains more than 400 differentially expressed genes). In both of the filtered simulation conditions, all of the methods get close to a precision of 0.1 or 0.2 when 400 markers are selected, and all of the curves are still decreasing at this point (a precision of 0.1 corresponds to random gene selection on these data sets). There are around 2000 differentially expressed genes in the un-filtered simulation condition, so the fact that the precision drops significantly when selecting up to 400 markers indicates proportionally similar behavior to the filtered data sets.

**Fig. 13:**
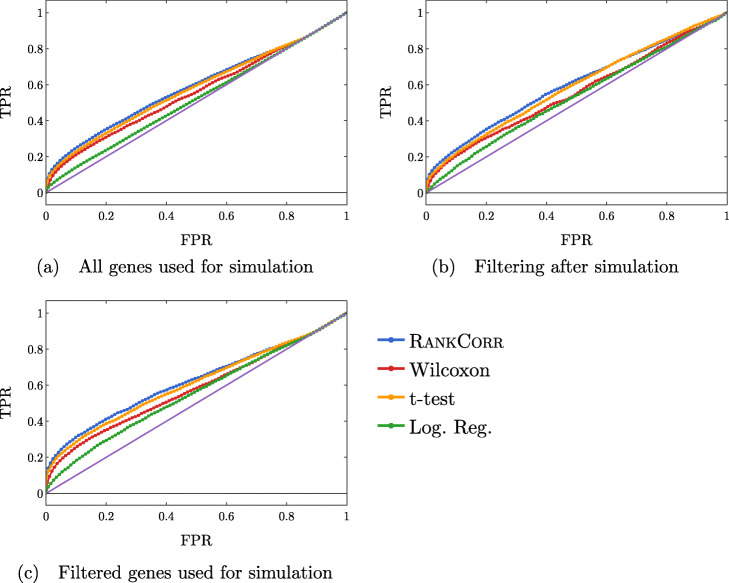
ROC curves. Each sub-figure corresponds to a simulation method and the four lines correspond to the different marker selection algorithms. The solid (purple) line is the diagonal TPR = FPR

The ROC curves in Fig. 13 also reflect this behavior: the curves increase (above the diagonal) quite rapidly for a short period of time, but then remain close to the diagonal overall.

These data are somewhat expected: in the simulations that come from all of the genes, many of the “differentially expressed” genes show low levels of expression. Thus, we would expect that the ROC curves should end up close to the diagonal as intermediate to large numbers of total markers are selected (since finding these low expression markers should be close to random selection). The filtered data sets could have solved this problem; however, the filtering method used here (see the full synthetic data description in the Methods) preserves the relative proportions of low- and high-expression genes and (possibly for this reason) do not affect the ROC curves very much.

Another explanation for these difficulties could be the differential expression parameters used in the Splat simulation. With these default parameters, the gene mean for some of the “differentially expressed” genes are only slightly different between the two clusters (for specifics, see the synthetic data discussion in the Methods). Thus, although the simulation may label these genes as differentially expressed, detecting the differential expression by any method will be very difficult. This underscores the differences between biological markers and differentially expressed genes: these differentially expressed genes would not be good practical biological markers, as it would be very difficult to tell two clusters apart based on the expression levels of these types of genes without collecting a lot of data.

Regardless, both of these plots support the notion that the methods are able to easily identify a small set of differentially expressed genes from the synthetic data but then rapidly start to have difficulties as more genes are selected. In addition, the RANKCORR method consistently shows the highest value of precision and TPR.

#### Inconsistent results are obtained when these simulated genes are filtered by dispersion

Comparing Fig. 12a-c across the simulation conditions, we see that the highest precision for each of the marker selection methods is obtained by using all genes for simulation, without any filtering. It is tough to explain why filtering genes by dispersion (the filtering method that we use here; see the synthetic data discussion information in the Methods) after simulating produces lower precision scores than not filtering. Since the t-test (for example) works by choosing genes based on a *p*-value score, and the genetic information is not changed by the filtering process (*p*-values would be the same in both the unfiltered and filtered after simulation data sets), it must be the case that many of the differentially expressed genes are removed from the data set when we filter after simulation. The highly variable genes selected by the filtering method used here are not required to have high expression; thus, there is no obvious reason that many differentially expressed genes should be filtered out.

Note that a similar effect is not observed in the ZHENG data sets (see Figs. 8 and 9), suggesting that this inconsistency is an artifact of the simulation methods used here. Simulating scRNA-seq data is itself a difficult task; see also [32] for a further discussion of the difficulties involved in simulating scRNA-seq data (and a tool that can help to expose these types of issues). Nonetheless, filtering genes is quite a heuristic process, and there is still more work to be done in fully understanding how this filtering impacts real scRNA-seq data^3^. At the very least, it is clear that the process of filtering genes by dispersion does not commute with the simulation methods used here, since filtering before simulation shows higher precision than filtering after simulation.

#### The classification error rate is an informative but coarse metric

**Fig. 14:**
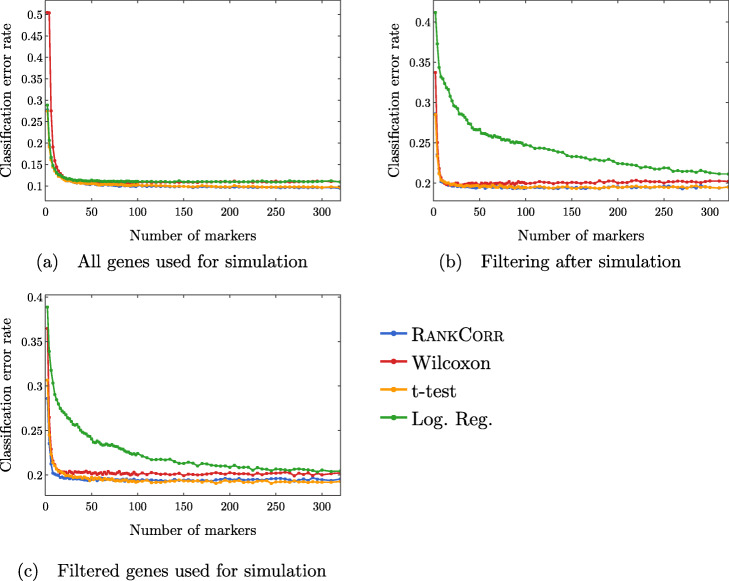
Clustering error rates using the Random Forest classifier for the first 500 markers chosen by each method. The sub-figures correspond to different simulation conditions. The RANKCORR algorithm consistently produces the smallest values of the clustering error rate

Finally, we examine the classification error rate of the methods applied to the synthetic data in Fig. 14. It is interesting to note that, with only two clusters, we still misclassify a minimum of around 10% of cells. This suggests that the simulated data are not well separated - the differential expression introduced in the synthetic data is not strong enough to easily separate the two clusters. Moreover, apart from the curves corresponding to the logistic regression method, all of the curves look to be fairly constant after a small number of markers have been selected (approximately 50 for the simulations based on all genes and approximately 30 for the simulations based on filtered data). This further supports the discussion from above - the methods start by quickly choosing a small number of good markers; after this, the genes that are selected do not provide significantly more information about the clustering.

Note that the methods that show higher precision in Fig. 12 also show a lower classification error in Fig. 14. On the other hand, logistic regression shows poor precision levels on the filtered data sets and also appears significantly worse than the other methods in the classification error rate curves. Thus, according to these experiments, the classification error rate seems to be a coarse but reasonable measure of how well a set of markers describes the data set. In this example, if one methods performs worse than another method according to the classification error rate curves (Fig. 14), then the same relationship holds in the precision curves (Fig. 12). Some large differences in precision are eliminated in the classification error rate curves, however, and thus the classification error rate should be considered with a grain of salt. That is, the classification error rate is informative, but it does not provide a full statistical picture of how the methods are actually performing.

## Discussion

### The difficulties of benchmarking and the importance of simulated data

Benchmarking marker selection algorithms on scRNA-seq data is inherently a difficult task. The lack of a ground truth set of markers requires for us to devise performance evaluation metrics that will illuminate the information contained in a selected set of genes. We have examined several natural evaluation metrics in this work; these metrics sometimes produce conflicting results, however. Our experiments make it clear that these metrics provide different ways to view the information contained in a set of genes rather than capturing the full picture provided by of a set of markers.

Having a ground truth set of markers available makes the evaluation of marker selection algorithms much more explicit. In the synthetic data here, for example, it becomes apparent that the methods rapidly select a set of markers that provide a lot of information about the clustering, then essentially start picking things by chance. This type of behavior can only be revealed by a study with a known ground truth.

On the other hand, simulating scRNA-seq data is itself a difficult problem. The simulated data that we consider in this work behaves strangely when we filter it by selecting highly variable genes. In particular, the filtering process considered here seems to remove many of the useful differentially expressed genes in the simulated data. This type of behavior was not observed in the ZHENGFILT experimental data set, where working only with high variance genes had little impact on the marker set evaluation metrics. Better simulation methods, and mathematical results formalizing the quality of simulated data, are extremely important future projects. See [32, 33] for some work towards these goals.

### The relationship between marker selection and the process of defining cell types

The marker selection framework considered in this work is quite narrow. It is focused on discrete cell types, and (as shown in the PAUL data set) does not handle cell differentiation trajectory patterns very well. Moreover, we assume that the genetic information that we supply to a marker selection algorithm consists of cells that are already partitioned into cell types. This is consistent with the data processing pipeline that many researchers currently follow (cluster the scRNA-seq data with an algorithm, then find markers for the clusters that are produced [18, 19]); it seems more reasonable to allow for marker selection to help guide the process of finding and defining cell types, however.

For example, future marker selection methods could find markers that are useful for identifying certain regions of the transcriptome space (in an unsupervised or semi-supervised manner). This would allow for clarity along a cell differentiation pathway - at any point on the trajectory, a researcher could view the markers that identify the nearby area, and to what degree each marker identifies the area. Thus, cell types (or differentiation pathways) could be suggested based on marker genes. These cell types might themselves reveal more informative markers, creating an iterative process: let the markers guide the clustering and vice versa. Such a method is known as an *embedded* feature selection method in the computer science literature; adapting an embedded feature selection method to scRNA-seq data is left for future consideration.

## Conclusions

Across a wide variety of data sets (large and small; data sets containing cell differentiation trajectories; datasets with well separated clusters; biologically defined clusters; algorithmically defined clusters) and looking at many different performance metrics, it is impossible (and even inappropriate) to say that any of the methods tested selects better markers than all of the others. Indeed, the marker selection method that was “best” depended on the data set as well as the evaluation metric in question, and the difference in performance between the “best” marker selection algorithm and the “worst” was often quite small.

Thus, the major factors that differentiate the methods examined in this work are the computational resources (both physical and temporal) that the methods require. Since the algorithms show similar overall quality, researchers should prefer marker selection methods that are fast and light.

In addition to this, as technology advances, the trend is towards the generation of larger and larger data sets. High throughput sequencing protocols are becoming more efficient and cheaper, and other statistical and computational methods are improved when many samples are collected. Through imputation and smoothing methods (see e.g. [34–36]), a detailed description of the transcriptome space can be revealed even when low numbers of reads are collected in individual cells. Thus, the speed of a marker selection algorithm will only become more important.

The RANKCORR, Wilcoxon, t-test, and logistic regression methods run the fastest of all of the methods considered in this work. They run considerably faster and/or lighter than any of the complex statistical methods that have been designed specifically for scRNA-seq data. Logistic regression does not scale particularly well with the data set size, however, and it requires an amount of resources that is not competitive with the other three methods on the largest data sets. Moreover, logistic regression exhibits poor performance on several of the data sets considered in this work, especially when selecting small numbers of markers. Thus, as a general guideline, RANKCORR, Wilcoxon, and the t-test are the optimal marker selection algorithms examined in this work for the analysis of large, sparse UMI counts data. This recommendation is further bolstered by the fact that these three algorithms tend to perform well in the experiments that we have considered here, especially when selecting lower numbers of markers.

The RANKCORR algorithm, introduced in this work, is the slowest of the three recommended algorithms. Nonetheless, RANKCORR outperformed the other fast algorithms in our synthetic tests. In addition, it provides some interpretability in the multi-class marker selection scenario. Specifically, RANKCORR attempts to select an informative number of markers for each cluster (rather than just a fixed number for each cluster), generally selecting more markers for clusters that we are less certain about. The work of properly selecting sets of markers in a multi-class scenario has not been completed, however, and RANKCORR only proposes one step. Overall, as a fast and efficient marker selection algorithm, RANKCORR is a valuable addition to the set of scRNA-seq analysis tools.

RANKCORR also involves taking a rank transform of scRNA-seq counts data. The rank transformation has other uses in scRNA-seq; it is thus useful to understand the further properties of the rank transformation. These properties will be explored in upcoming work.

Finally, in the way that data processing pipelines are currently set up, researchers will often be forced to select markers without the knowledge of a ground truth set of markers. Thus, it may be valuable to consider metrics such as the ones discussed in this work when performing marker selection. Combining the values of several of the metrics may help to aid researchers in deciding when they have selected enough markers to adequately describe their cell types (so that they are not considering genes that were chosen at random), for example. The question of how to stop selecting markers is another important consideration for future work.

## Methods

### Details of the RANKCORR algorithm

Given a vector *x*∈***R***^*p*^ and a parameter *β*∈***R***, we define the soft-thresholding operator *T*_*β*_(*x*):***R***^*p*^→***R***^*p*^ by

4 $$ T_{\beta}(x)_{j} = \left\{\begin{array}{lll} \text{sign}\left(x_{j}\right)\left|x_{j} - \beta \right| &\colon & \left|x_{j}\right| > \beta\\ 0 & \colon & \text{otherwise} \end{array}\right.  $$

We say that *T*_*β*_(*x*) is a soft-thresholding of the vector *x*.

#### Setup

Recall the notation from the Results: let *X*∈***R***^*n*×*p*^ be a scRNA-seq count matrix (*n* cells, *p* genes). Label the cells with the numbers in [ *n*]. Given a subset *S*⊂[ *n*] of cells, define *τ*∈{±1}^*n*^ such that *τ*_*i*_=+1 if cell *i* is in the subset (that is, if *i* is in *S*) and *τ*_*i*_=−1 otherwise. We refer to *τ* as the *cluster indicator vector* for the set *S*.

To find markers for *S*, we desire a vector *ω*∈***R***^*p*^ such that

5 $$ \tau = \text{sign}\left(\overline{X} \omega\right)  $$

where $\overline {X}$ denotes a transformed version of *X* (we use the specific transformation (2) for RANKCORR). Note that, if cell *i* is in *S*, then $\left \langle \overline {x}_{i}, \omega \right \rangle >0$; otherwise, $\langle \overline {x}_{i}, \omega \rangle < 0$. Thus, *ω* is the normal vector to a hyperplane passing through the origin that separates the cells that are in *S* from all other cells. In this framework, the nonzero entries of *ω* are marker genes for the subset *S* - they are the features that separate the given cell type from the other cells. To obtain a small number of markers, we desire a sparse solution *ω*; that is, a solution *ω* with few nonzero entries.

Unfortunately, it is computationally infeasible to find the sparsest vector *ω*^∗^ that satisfies (5) (see [37]). In addition, for noisy experimental data, there is probably no vector *ω* that will perfectly satisfy (5).

In [15], the authors circumvent these issues by assuming that there is a vector *ω* (and a value *t* such that ∥*ω*∥_0_≤*t*) that *mostly* satisfies (5) (i.e. the vector equality does not need to hold in all coordinates). They present the convex optimization (3) that uses $\overline {X}, \tau $ and an input sparsity parameter *s* to produce an approximate solution $\hat {\omega }$ that is “close” (in a technical sense) to this true sparse *ω*. We refer to *s* as a sparsity parameter due to the fact that it influences the number of zeros in the approximation $\hat {\omega }$. Specifically, *s* controls the size of the set that the approximation $\hat {\omega }$ will be chosen from: when *s*≥*t* (so that the true signal *ω* has ∥*ω*∥_0_≤*s*), then *ω* will be in the feasible region of the optimization. For convenience, the optimization is reproduced below.

6 $$ \begin{aligned} \hat{\omega} =\underset{\omega} {\arg\min} & \sum\limits_{i=1}^{n} \tau_{i} \langle \overline{x}_{i}, \omega \rangle\\ \mathrm{subject\ to\ } & \left\|\omega\right\|_{2} \leq 1, \left\|\omega\right\|_{1} \leq \sqrt{s} \end{aligned}  $$

In the optimization (3), $\overline {x}_{i}$ denotes the *i*-th row of $\overline {X}$.

A couple of technical points deserve mention here: first, there are other efficient methods for obtaining an approximate solution $\hat {\omega }$ to (5). As mentioned in the overview of RANKCORR, however, the optimization (3) has previously [14] been developed into SPA, a feature selection algorithm for use with sparse biological data (specifically, mass spectrometry data in proteomics). We thus focus on the optimization (3) to solve (5) for this work.

Moreover, there are algorithms for finding general sparse separating hyperplanes (e.g. sparse support vector machines, see Section 4.5 of [8]). These methods don’t assume that the hyperplane passes through the origin, but they are somewhat slow, and it would not be reasonable to use them with the massive scRNA-seq data sets that are considered in this paper. Thus, to develop a fast marker selection method, we keep the additional assumption that the separating hyperplanes pass through the origin.

Finally, the fact that we are searching for a hyperplane passing through the origin necessitates a good choice of the transformation that yields $\overline {X}$ from *X*. For example, if *X* is left unchanged, then all of the cells lie in the first orthant of ***R***^*p*^. In this case there is almost certainly no *ω* that satisfies (5); many hyperplanes, for example, do not even pass through the first orthant.

In SPA, the authors of [14] construct the input $\overline {X}$ by “quasi-standardizing” the columns of the data matrix *X*: the column $\overline {X}_{j}$ is a linear combination of the centered version of *X*_*j*_ and the standardized version of *X*_*j*_; that is,

7 $$ \overline{X}_{j} = \alpha^{2\left(1 - \left|\rho_{j}\right|\right)}\cdot\lambda \cdot \left(X_{j} - \mu\left(X_{j}\right)\right) + \left(1-\alpha^{2\left(1-|\rho_{j}|\right)}\right) \cdot\left(\frac{X_{j} - \mu\left(X_{j}\right)}{\sigma\left(X_{j}\right)}\right)  $$

where *α* and *λ* are hyperparameters and *ρ*_*j*_ is the empirical correlation of *X*_*j*_ with *τ*.

The goal of the quasi-standardization in SPA is to provide more weight to the genes that are highly correlated with the labels *τ* (regardless of their expression levels) while also downweighting high expression genes that are not well correlated with the labels *τ* (that is, *λ* should be quite large). Similar behavior is accomplished in RANKCORR through the use of the rank transformation, see the intuitive description of the rank transformation in the Background for more information. Thus, RANKCORR has the benefit that there are no hyperparameters to tune. In experiments, we see that RANKCORR runs much more quickly than the method SPA from [14] and generally produces better results on scRNA-seq data.

#### A fast algorithm for solving the optimization (3)

Given a matrix $\overline {X}$ and a signal *ω* (with ∥*ω*∥_0_≤*s*), let $\tau = \text {sign}(\overline {X}\omega)$. Recall that the optimization (3) uses $\overline {X}, \tau $, and *s* to provide an approximation $\hat {\omega }$ that is “close” to *ω*.

In both [38] and [39], the authors show that the solution $\hat {\omega }$ to (3) is given by a normalized soft thresholding of the vector

8 $$ v = \sum\limits_{i=1}^{n} \tau_{i} x_{i},  $$

where *x*_*i*_ represents the *i*-th row of $\overline {X}$. That is,

9 $$ \hat{\omega} = T_{\beta}(v)/\left\|T_{\beta}(v)\right\|_{2},  $$

where *β* is a parameter that depends on *s* and the cluster indicator vector *τ* in a non-trivial manner. For feature selection, we are interested only in the support of $\hat {\omega }$. Thus, the optimization (3) can be solved simply by soft-thresholding at each coordinate of *v*. Algorithm SELECT implements this idea to quickly find the support of $\hat {\omega }$. It is defined in Algorithm 1.


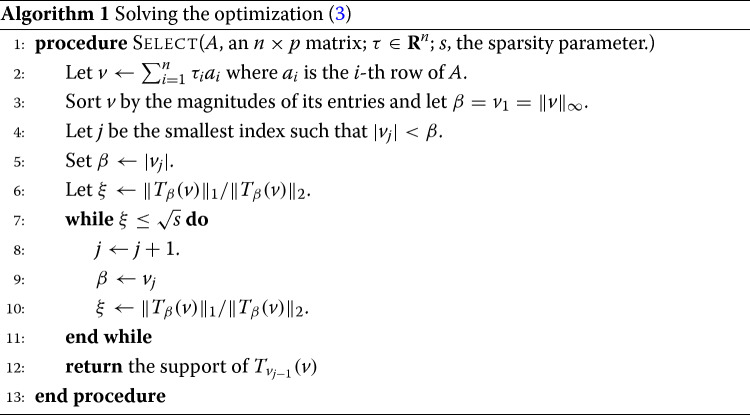


Intuitively, Algorithm 1 works in the following manner. For *s*>1, note that the feasible set $\left \{x \in \boldsymbol {\mathrm {R}}^{p} \colon \left \|x\right \|_{1} \leq \sqrt {s}, \left \|x\right \|_{2} \leq 1\right \}$ looks like the set $\left \{x \in \boldsymbol {\mathrm {R}}^{p} \colon \left \|x\right \|_{1}\leq \sqrt {s}\right \}$ with the corners chopped off and rounded. Thus, we start by creating $\tilde {v}$, a normalized non-zero soft-thresholding of *v* that has as few nonzero entries as possible^4^. We then soft-threshold *v* by smaller and smaller values so that $\tilde {v}$ gains more non-zero coordinates and thus points further away from a coordinate axis. Since $\tilde {v}$ is always normalized, it is on the 2-sphere {*x*:∥*x*∥=1}. Thus, we can stop when $\tilde {v}$ is also on the 1-sphere $\left \{x \colon \left \|x\right \|_{1} = \sqrt {s}\right \}$. Then, $\tilde {v}$ is a point where the 1-sphere intersects the 2-sphere, and the support of this $\tilde {v}$ are the features that we are interested in selecting.

There is also a faster algorithm for solving a problem that is equivalent to (3) presented in [39]. This algorithm does not allow for an interesting generalization to the multi-class problem, however.

We use SELECT in our implementations of both SPA and RANKCORR; this also means that our implementation of SPA is faster than it would have appeared in past work, including [14] (where SPA is introduced).

#### Applying SELECT to rank transformed data

This section contains an algorithm RANKBIN (defined in Algorithm 2) that uses SELECT along with the rank transformation to select markers for a fixed cell type in a way that is motivated by SPA. The inputs are a UMI counts matrix *X*∈***R***^*n*×*p*^, a vector *τ*∈{±1}^*n*^, and a sparsity parameter *s*. Note that this is still a binary marker selection method, since the entries of *τ* are either +1 or −1. The extension to the multi-class case is described in the next section.


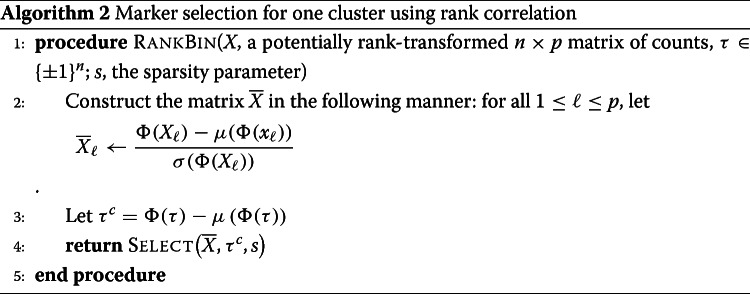


In Algorithm 2, the construction of $\overline {X}$ is motivated by the quasi-standardization (7) of the data matrix *X* in SPA. In RANKBIN, the columns of the data matrix *X* are standardized *after* they are rank transformed. Moreover, motivated by the work in [14] and [38], the vector *τ* is replaced with *Φ*(*τ*)−*μ*(*Φ*(*τ*)) in RANKBIN. That is, the rank transformation is applied both to the data matrix *X* and the class indicator *τ*. In this case, the vector *v* that we soft threshold when we call SELECT (see (8), above) has entries given by

10 $$ v_{j} = \sum\limits_{i=1}^{n} \left(\Phi(\tau)_{i} - \mu(\Phi(\tau))\right) \frac{\Phi\left(X_{j}\right)_{i} - \mu\left(\Phi\left(X_{j}\right)\right)}{\sigma\left(\Phi\left(X_{j}\right)\right)}.  $$

That is, entry *j* of *v* is (proportional to) the Spearman rank correlation between gene *j* and the vector *τ*. Thus, RANKBIN will select the genes that have the highest (absolute) Spearman correlation with the vector of class labels. It is possible to show that replacing *τ* with *Φ*(*τ*)−*μ*(*Φ*(*τ*)) has no effect on the markers that are selected by the algorithm; algorithm RANKBIN is written with $\overline {\Phi (\tau)}$ instead of *τ* to emphasize the connection with the Spearman rank correlation. (Similar calculations show that the markers selected by SPA are generally those that have high correlation with the cell type labels.)

Tangentially, note that the rows of the rank transformed and standardized data matrix $\overline {X}$ in Algorithm 2 (the RANKBIN algorithm) come from a bounded - and thus sub-Gaussian - distribution with mean 0 and variance 1. Thus, this matrix $\overline {X}$ matches many of the hypotheses of the theoretical guarantees about the solution to (3) that are presented in [40] (the rows of $\overline {X}$ are not independent, however).

#### RANKCORR: multi-class marker selection

RANKCORR, defined in Algorithm 3 works by fixing a parameter *s* and applying RANKBIN to each of the cell types in the data set. Specifically, fix a sparsity parameter *s*; this parameter will be the same for all of the cell types. For cell type *j*, construct the vector *τ*^*j*^ with $\tau _{i}^{j} = 1$ if cell *i* is in cell type *j* and $\tau _{i}^{j} = -1$ otherwise. Then run RANKBIN on the data matrix *X*, *τ*^*j*^, and the fixed sparsity parameter *s* to get the markers for cell type *j*. This will usually result in a different (informative) number of markers selected for each cell type.


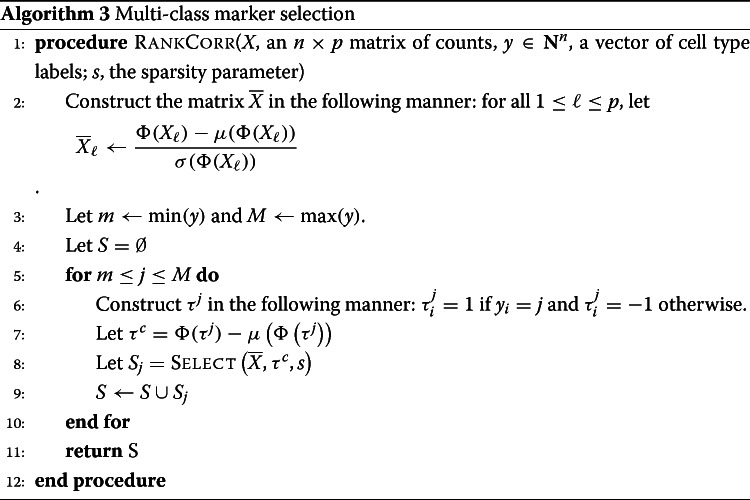


Note that the computation of $\overline {X}$ does not depend on the group *j* that we are selecting markers for; thus, $\overline {X}$ can be computed one time at the start of marker selection. To explicitly avoid this extra computation, we write RANKCORR without calling RANKBIN; the ideas behind the steps in the loop are provided in our discussion of RANKBIN, however.

In RANKCORR, we take the union of all the markers selected for each cluster to get a set of markers that will represent all of the given cell types. This step is to allow for easier collection of benchmarking statistics - we would like to capture how well a selected set of markers informs us about an entire clustering. In practice, the sets of markers could be kept separate to give information about individual cell types. Note that there could still be duplicate markers in these sets - here, we do not address the problem of dealing with duplicates in a smart way.

As a final note, when we are not interested in a full set of markers, we can quickly compare two genes to determine the gene that is favored by RANKCORR for a fixed cluster *q*: gene *j* will be chosen as a marker more favorably than gene *k* if the norm of the Spearman correlation between *τ*^*q*^ and *X*_*j*_ is larger than the norm of the Spearman correlation between *τ*^*q*^ and *X*_*k*_.

### Marker evaluation methods for experimental data

Below, we discuss two general procedures for the evaluation of a set of markers: supervised classification (that incorporates the given ground truth clustering as prior information) and unsupervised clustering (that does not).

Assuming that the data set contains *n* points in *k* clusters, the result obtained by either classifying or clustering the data is a vector of predicted cell type labels $\hat {y} \in \boldsymbol {\mathrm {Z}}^{n}$. We would like to compare this to the “ground truth” cluster label vector *y*∈[*k*]^*n*^. The full information about the similarity between *y* and $\hat {y}$ can be presented in terms of a confusion matrix; this is unwieldy when many such comparisons are required, however. For this reason, many summary statistics have been developed in the machine learning literature for the classification [22] and clustering [23] settings. We choose to examine several of these metrics in this work; the full list is summarized in Table 3.

#### Cross validation

In order to avoid overfitting, we perform all marker selection, classification, and clustering using 5-fold cross validation. See Additional file 1, Figure 1 for a summary of this procedure. Specifically, we split the cells into five groups (called “folds”). For each fold, we combine the other four folds into one data set, find the markers on the dataset containing four folds, and train the classifier using the selected markers on the dataset containing four folds as the training data. We then apply the trained classifier to the initial (held-out) fold and perform clustering on the initial fold using the markers that were selected on the other four folds. In this way, the initial fold is “test data” for the classifier/clustering metrics.

Repeating this process for all five folds creates a classification for the entire data set. On the other hand, we get a separate clustering for each fold, and these clustering solutions may be incompatible (they may contain different numbers of clusters, for example). See the section on clustering evaluation metrics for how we reconcile this.

#### Supervised classification

In order to incorporate information about the ground truth clustering into an evaluation metric, we train a multi-class classifier on the scRNA-seq data using the cluster labels as the target output. In order to evaluate the selected marker genes, we train the classifier using only the marker genes as the input data. When applied to a vector of counts (e.g. the counts of the markers in a cell), the classifier outputs a prediction of the cluster that the vector belongs to.

**Training a classifier.** The specifics of how a classifier is trained are presented in Algorithm 4.


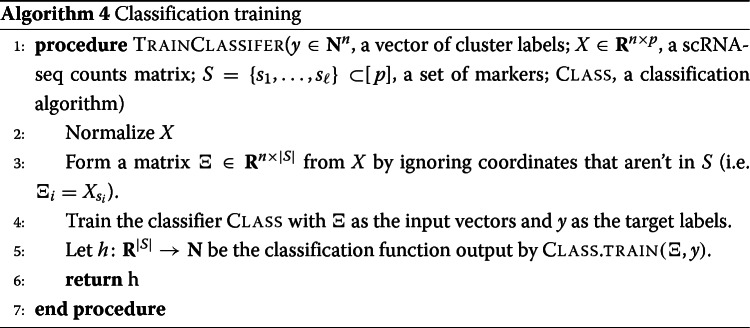


In line 2 of Algorithm 4, we normalize the matrix *X*. It is possible to use any normalization for this step; for the purposes of our analysis we use a log normalization procedure that is commonly found in the scRNA-seq literature. Specifically, we perform a library-size normalization so that the sum of the entries in each row of *X* is 10,000 and follow this by taking the base 2 logarithm of (1 plus) each entry of *X* to create a “log normalized” counts matrix.

Library size normalization was introduced in [41] to account for differences in capture efficiency between cells and taking a logarithm has it roots in bulk RNA-seq where it is used to attenuate technical variance (see [42]). Since log normalization of this type is often applied when clustering scRNA-seq counts data in a data processing pipeline, we apply log normalization when attempting to recover the information in the given clusters. It is important to note that the marker selection algorithms that we examine in this work do not assume that the input counts data are normalized (apart from when noted in their descriptions).

**Classification evaluation metrics.** We select markers and classify the cells using 5-fold cross-validation (see also Additional file 1, Figure 1). Once we have classified all cells in the data set, we examine how well the vector of classification labels matches the vector of ground truth cluster labels. Since we are in a classification framework, we use multi-class classification evaluation metrics for this purpose. In particular, we examine the classification error (1- accuracy) and precision of the classification compared to the known ground truth. For precision in a multi-class setting, we compute the precision for each class (as in a binary classification setting) and then take a weighted average of the per-class precision values, weighted by the class sizes. Finally, we also examine the Matthews correlation coefficient, which is a summary statistic that incorporates information about the entire confusion matrix. See [22] for more information about these statistics.

In all of the tests that we perform in this work, the precision and Matthews correlation coefficient curves look subjectively similar to each other (though the actual values of the statistics do differ), while the classification error appears very similar to the other curves except it is flipped upside down. It is not clear why these summary statistics look as similar as they do. In any case, we generally only present the classification error rate in this document; the precision and Matthews correlation coefficient pictures can be found in Additional file 1 (Figures 2 and 3 for ZEISEL; Figures 5 and 6 for PAUL; Figures 8–11 for ZHENGFILT and ZHENGFULL; and Figure 13 for 10XMOUSE).

**Classifiers** We examine two classifiers to evaluate the marker sets (so that we are computing two classifications for each selected set of markers, and looking at all three metrics for both classifications).

The first is a simple (and fast) nearest centroids method that uses information about the original clustering to determine the locations of the cluster centroids. We refer to this as the Nearest Centroids Classifier (NCC). See the end of this section for a full description of the NCC. In the second, we use the Random Forest Classifier (RFC) that is implemented in the Python package scikit-learn ([30]), version 0.20.0, with nestimators = 100.

The summary statistics of the classifications produced when using the RFC are always better (more optimal) than the statistics that are produced when using the NCC. In addition, the overall shape of the curves produced using the RFC mostly mirror the curves produced using the NCC. The RFC is too slow to run on the largest data sets that we examine for testing. Since the RFC and NFC curves look similar for the smaller data sets, we are not concerned that we are missing information here.

Also note that, even with nestimators = 100, there is a significant amount of variability in the classification results obtained through the RFC. That is, running the RFC multiple times with the same set of markers will produce different classification results. See Additional file 1, Figure 15 for a visualization of the differences in error rate that can be obtained when running the RFC twice on the same sets of markers (this example is created using the PAUL data set; see the discussion of experimental data sets).

**The nearest centroids classifier (NCC).** The NearestCentroids.train method is presented in Algorithm 5.


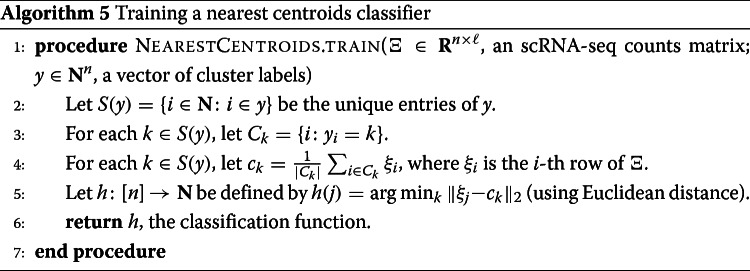


#### Unsupervised clustering

Another natural way to measure the information in a selected set of markers is to cluster the data using only the selected coordinates in an unsupervised manner and compare this new clustering to the original clustering. Clustering scRNA-seq is itself a complicated problem that has inspired a great deal of study; here we restrict ourselves to Louvain clustering as implemented in the scanpy (version 1.3.7) package. Louvain clustering was introduced for use with scRNA-seq experiments in [43] and it is currently the recommended method for clustering scRNA-seq data in several commonly-used software packages including scanpy [5] and Seurat [6].

**The clustering procedure.** A method for performing Louvain clustering on scRNA-seq data is presented in Algorithm 6. Note that we do not perform any dimensionality reduction (e.g. PCA) before finding nearest neighbors or performing the clustering. This is due to the fact that we project the data onto the selected markers. These markers are meant to capture the important dimensions in the data - they are the features that have the most information about the clustering according to a marker selection algorithm. Thus, we work in the space spanned by these markers without performing any additional dimensionality reduction.


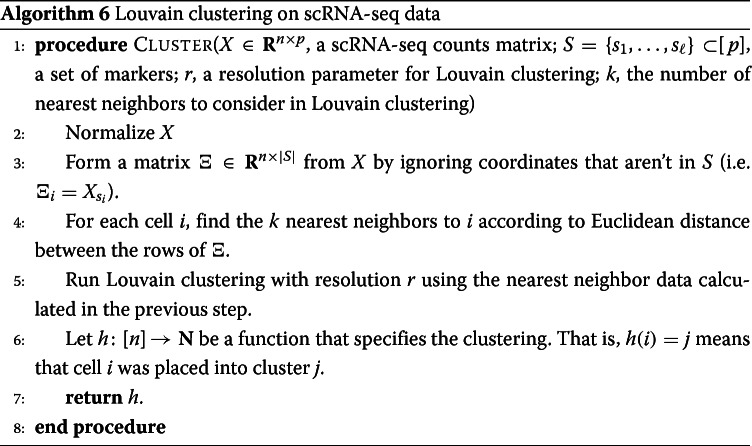


In line 2, of Algorithm 6, we normalize the counts matrix *X*. As in the case of the supervised classification metrics, we apply log-normalization for this step.

**Clustering evaluation metrics.** The unsupervised clustering is compared to the ground truth clustering using three metrics from the machine learning literature: the Adjusted Rand Index (ARI), Adjusted Mutual Information (AMI), and the Fowlkes-Mallows score (FMS). All three of these scores attempt to capture the amount of similarity between two groupings of one data set (e.g. the unsupervised clustering produced using a selected marker set and the ground truth clustering). They are also normalized scores: values near zero indicate that the cluster labels are close to random, while positive values indicate better performance. All of the scores have a maximum value of +1. Moreover, all three of these metrics do not make any assumption about the number of clusters: the unsupervised clustering can have a different number of clusters from the ground truth clustering and these indices can still be computed. See [23] for more information about these metrics.

We again use 5-fold cross-validation to compute the clustering performance metrics. Note that the clustering solutions for the different folds may be incompatible: for example, the number of clusters in the Louvain cluster solution for the first fold may be different from the number of clusters in the Louvain cluster solution for the second fold, and there may be no obvious way to relate the clusters in the first fold to the clusters in the second fold. For this reason, we compute the clustering performance metrics separately on each fold, comparing the Louvain cluster solution to the ground truth clustering restricted to the fold. The scores that we report are averaged over all of the folds (and when we optimize over the resolution parameter *r*, discussed below, we find the optimal value of the average over the folds).

Note that some of the fine structure from the ground truth clustering may not be maintained in a specific fold and thus it is impossible to capture this structure when performing Louvain clustering on the fold. This means that the actual values of these metrics are not particularly informative - it is more useful to compare the different methods along a metric. In addition, in all of the Louvain clusterings for a specific data set, we fix the value of *k*, the number of nearest neighbours that we consider. Thus, small differences between the scores are not particularly informative, as they could disappear if *k* was selected perfectly for each method. Nonetheless, it is useful to get an idea as to how well the markers selected by different algorithms could be used in an unsupervised manner to recover a given clustering.

**Choice of Louvain clustering parameters.** Louvain clustering requires the input of a number *k* of nearest neighbors and a resolution parameter *r*. It would be ideal to optimize both *k* and *r* for each set of markers on each data set for each clustering comparison metric; then we would be comparing the “optimal” performances of the marker selection algorithms under each metric. This is not computationally realistic for all of the data sets in consideration here, however.

Thus, for a given data set, we fix the value of *k*. For each set of markers on the data set, we compute the *k* nearest neighbors (once), and then quickly optimize over the resolution parameter *r*. To optimize *r*, we examine a grid from *r*=0.1 to *r*=3.0 with a step size of 0.1. This allows us to compute approximately optimal values of each of the metrics for each set of selected markers in a computationally efficient manner. Importantly, the resolution parameter is optimized for each metric using each marker selection algorithm.

**Choosing*k*.** On the PAUL and ZEISEL data sets, we examined values of *k* varying from 15 to 30 (with a step size of 5). For each value of *k*, we used the RANKCORR algorithm to optimize over *r* (varying from *r*=0.1 to *r*=3.0 with a step size of 0.1), and we varied the number of markers selected to get a picture of the entire parameter space. The curves that are produced by this process are quite similar for different values of *k* (see Additional file 1, Figures 16 and 17). The value of *k* is chosen to be the one that subjectively appears to optimize the performance of the majority of the metrics.

On the ZEISEL dataset, it appears that *k*=15 nearest neighbors does not capture quite enough of the cluster structure, while *k*=30 nearest neighbors results in lower scores than *k*=25. We thus fix *k* at 25 for the unsupervised clustering evaluation on the ZEISEL data set. See Additional file 1, Figure 16 for the data that were used for this determination.

For the PAUL data set, we observed that changing the number of nearest neighbors used in the Louvain clustering has little effect on the ARI, AMI, or FM scores. It appeared that the scores were slightly improved for *k*=30 when small numbers of markers were selected, thus we fixed *k* at 30 for the PAUL data set. See Additional file 1, Figure 17.

The ZHENGFULL and ZHENGFILT data sets are large, and thus we focus on the ZHENGFILT data set when considering the unsupervised clustering metric. To estimate a value of *k*, the fixed number of nearest neighbours that we use for all of the clusterings, we computed a Louvain clustering that looks quite similar to the bulk labels in a UMAP plot. This clustering used 25 nearest neighbors (and used the top 50 PCs); thus we fix *k* at 25 for the ZHENG data sets. See Additional file 1, Figure 18 to see a comparison of the bulk labels and the generated Louvain clustering in UMAP space.

### Experimental data sets

We examine four publicly available experimental scRNA-seq data sets in this work. We focus on data sets that have been clustered, with clusters that have been biologically verified in some way. In addition, we mostly examine data sets that were collected using microfluidic protocols (Drop-seq, 10X) with UMIs. This is due to the fact that these protocols tend to collect a smaller number of reads in a larger number of cells (producing large amounts of sparse data). These data sets are summarized in Table 1. We discuss them further below. See the statement on data availability for how to obtain these data and for more information about the scripts used for pre-processing.

ZEISEL. We work with one well-known reference fluidigm data set. This is ZEISEL, a data set consisting of mouse neuron cells that was introduced in [24]. Neuron cells are generally well-differentiated, and thus this data set contains distinct clusters that should be quite easy to separate. In [24], the authors have additionally used in-depth analysis with known markers in combination with a biclustering method of their own design to painstakingly label each cell with a specific cell type. This labeling is the closest to an actual ground truth clustering of a dataset in the scRNA-seq literature - this fact makes ZEISEL a valuable data set for our benchmarking purposes.

For our ground truth clustering, we consider only the nine major classes that the authors define in [24]. In addition, we pre-process the data set using the cell_ranger flavor of the filter_genes_dispersion function in the scanpy Python package after library size normalization. We ask for the top 5000 most variable genes; the filter_genes_dispersion function only returns 4999 genes, however. We perform this pre-processing to speed up the marker selection process for the slower methods.

PAUL. The smallest data set that we examine is PAUL, a data set consisting of 2730 mouse bone marrow cells that was introduced in [25] and collected using the MARS-seq protocol. As opposed to ZEISEL, bone marrow cells consist of progenitor cells that are in the process of differentiating. Thus, there are no well separated cell types in the PAUL data - the data appear in a continuous trajectory. The authors of [25] define discrete cell types along this trajectory based on known markers, however: we consider this clustering from [25] to be the ground truth for our analysis in this manuscript.

ZHENG **data sets.** We perform an analysis of the data set introduced in [2] that consists of around 68 thousand human PBMCs from a single donor; we refer to this full data set as ZHENGFULL. These data were collected using 10x protocols.

The ground truth clustering for the ZHENG data set that we examine in this manuscript contains 11 clusters. It was constructed in [2] by maximizing correlation with purified reference cell populations. This clustering contains some cell types that should be easy to separate (e.g. B cells vs T cells), as well as some cell types that have mostly overlapping profiles (e.g. several types of T cells are included as different clusters). The cluster labels can be found on the scanpy_usage GitHub repository at https://github.com/theislab/scanpy_usage/blob/master/170503_zheng17/data/zheng17_bulk_lables.txt(we use commit 54607f0).

To be more precise, these clusters were determined in the following manner: first, the full data set was clustered (using k-means), and the clusters were assigned biological types based on known markers. The authors of [2] then took more cells (from the same donor) and isolated a set cells of each cell type that they found in their clustering of ZHENGFULL. They then sequenced the cells from the individual types. Finally, they used these pure samples to cluster the ZHENGFULL data set again: each cell is assigned to the type whose (average) profile correlates most strongly with the cell’s profile..

We additionally generate a data set ZHENGFILT from ZHENGFULL by restricting to the top 5000 most variable genes. We select the 5000 most variable genes by performing a library size normalization on the ZHENGFULL data set and then using the cell_ranger flavor of the filter_genes_dispersion function in the scanpy Python package. We can run the slower methods on ZHENGFILT.

**1.3 million mouse neurons.** Finally, we examine 10XMOUSE, a data set consisting of 1.3 million mouse neurons generated using 10x protocols [3]. As noted above, neurons are well-differentiated into cell types, so this data set should contain well-separated clusters. The “ground truth” clustering that we consider for this data set is a graph-based (Louvain) clustering performed on the full 10XMOUSE dataset by the team behind scanpy. It can be found from the scanpy_usage GitHub repository (https://github.com/theislab/scanpy_usage/tree/master/170522_visualizing_one_million_cells; we consider commit ba6eb85) As far as we know, this clustering has not been verified in any biological manner.

### Marker selection methods

A summary of the marker selection methods that we consider in this work is found in Table 2. In addition, we also implemented SCDE and D^3^E, but found these two methods to be too slow. We discuss our precise implementation details below. We use Python version 3.7 and R version 3.5.0 unless otherwise noted.

**Wilcoxon and the t-test.** The t-test and the Wilcoxon rank sum are general statistical methods that aren’t specifically designed for RNA-seq data, but they are still often used for the purposes of differential expression testing in the scRNA-seq literature. We use the Python scanpy package (version 1.3.7) implementation to find Wilcoxon rank sum and t-test *p*-values with some editing to the file _rank_genes_groups.py to fix several bugs (that are now fixed in the main release). See the data availability disclosure for how to find this edited file.

Both of these methods produce a score for each gene: when choosing the markers for the clusters, we use the absolute value of this score (so we would chose markers that have a large negative score as well). This is for more direct comparison to the RANKCORR method, where we choose markers by the absolute value of their coefficients. In addition, both of these methods correct the *p*-values that they produce using Benjamini-Hochberg correction. Finally, we use the version of the t-test in scanpy that overestimates the variance of the data.

**edgeR and MAST.** The methods edgeR and MAST were originally implemented in R. In order to run them with our existing framework, we use the rpy2 (version 2.9.4) package to access the methods through Python.

Based on the results and scripts from [7], edgeR (version 3.24.1) was run using the quasi-likelihood approach (QLF method) on the un-normalized scRNA-seq counts matrix *X*. For MAST (version 1.8.1), the data matrix *X* was normalized: the rows of *X* were scaled so that each row summed to 1 million (to approximate something that looks like “transcripts per million”) to create a scaled matrix *X*^*s*^ and then each entry $X^{s}_{ij}$ of *X*^*s*^ was replaced by $\log \left (X^{s}_{ij} + 1\right)$.

Again following [7], we ran both edgeR and MAST in two ways on the PAUL and ZEISEL data sets. In the first way, we only consider the cluster label when fitting the statistical model; these results are presented in the Results section above. For the second way, we additionally include the fraction of genes that are detected in each cell (“detection rate”) as a covariate. We refer to edgeR and MAST run the second way by edgeRdet and MASTdet respectively. According to the marker set evaluation metrics, edgeRdet and MASTdet perform similarly to the other methods considered in this work. In addition, edgeRdet (MASTdet) requires slightly more computational resources than edgeR (MAST). For this reason, we choose not to include the edgeRdet or MASTdet results in this manuscript; see Additional file 1, Figures 2–7 for that information.

**scVI.** scVI (version 0.2.4) is implemented in Python and utilizes GPUs for faster training. Although the authors provide evidence that their code can handle a data set with one million cells (scVI is tested on the 10XMOUSE data set in [29]), scVI requires steep computational resources - around 75 GB of RAM to go with one core and one GPU. We were unable to obtain this large amount of memory and a GPU at the same time, so we have been unable to reproduce their results here. One issue is that scVI does not work with sparse data structures (or it makes them dense after loading them); thus, it has been computationally infeasible for us to run scVI on the larger data sets like 10XMOUSE.

Another issue with scVI is that the differential expression methods included in the package are themselves computationally demanding (even after the model has been trained). As far as we can tell, requesting information about differentially expressed genes from a trained scVI instance produces a matrix of size larger than (10·*n*)×*p*, where *n* is number of cells and *p* is the number of genes in the original data set. Even restricting to the top 3000 variable genes in the 10XMOUSE data set, this matrix would require around 250 GB of memory to load into storage - in addition to the storage required for the (dense) 10XMOUSE dataset itself. Thus, although it may be possible to train the model on the 10XMOUSE data set, it will be nearly impossible with our computational resources to actually acquire the differential expression information from the trained model.

(An example of the extreme memory used by scVI: the ZEISEL dataset takes approximately 5 MB to store in a dense format. The matrix produced during the differential expression computation method requires 4.1 GB. The actual computation of the Bayes factors - the generalization of a *p*-value produced by scVI - uses a peak of 15-16GB of memory during processing. This high memory usage does not appear in the data presented in the Fig. 3 (in the Results section) since it is only required for post processing - actually training the scVI model does not require this memory.)

SPA. We examine the performance of the method SPA introduced in [14] and analyzed further in [38]. As discussed in the full description of RankCorr, SPA was the inspiration for this work, and selects markers based on a sparsity parameter *s*. SPA also has two hyperparameters that we are required to optimize over, and this causes SPA to take considerably longer than RANKCORR to run for a fixed value of *s*. Moreover, in a situation with no known ground truth, it is unclear what metric we would like to optimize when selecting these hyperparameters. For the current evaluation, we have minimized the classification error rate using the NCC (see information about the marker set evaluation metrics), but it is not clear that this would be the best metric to optimize in general. We choose the NCC classifier since the RFC exhibits some variance (see Additional file 1, Figure 15) - thus, optimizing the classification error rate according to the RFC classifier would produce an unstable set of markers (performing the optimization again would result in a different set of markers). We choose to optimize the supervised classification error (rather than one of the unsupervised clustering metrics) for the sake of speed - optimizing a slower evaluation metric would increase the computation time required for the SPA marker selection method.

Another inconvenience of the SPA method is that the hyperparameters affect the number of markers that are selected for a fixed value of *s*. This makes the number of markers selected by SPA method more inconsistent and unpredictable. For example, it has occurred that the “optimal” (in terms of minimizing the classification error rate using the NCC, as discussed above) choice of hyperparameters for sparsity parameters *s*_1_>*s*_2_ has resulted in a smaller number of markers selected for *s*_1_ than the “optimal” choice of hyperparameters for *s*_2_. That is, increasing *s* can lead to selecting smaller numbers of markers.

**Elastic nets.** The SPA method introduced in [15] is similar to an *L*_1_- and *L*_2_- regularized SVM without an offset (i.e. it finds a sparse separating hyperplane that is assumed to pass though the origin, the instinct for this is given near Equation (5) in the Methods). Thus, we also compare the performance of RANKCORR to that of the Elastic Nets version of LASSO: a least squares method with both *L*_1_ and *L*_2_ regularization [12]. Elastic Nets has two regularization parameters that need to be tweaked in order to find the optimal set of features; this requires extra cross-validation and therefore we are only able to run on the smaller PAUL and ZEISEL data sets. Although the scikit-learn (version 0.20.0) package contains a method for finding the regularization parameters by cross-validation, it still takes a significant manual effort in order to find a range of the regularization parameters that capture the full possible behavior of the system but will also allow for the objective function to converge (in a reasonable number of iterations) the majority of the time. The timing information presented in Fig. 3 only represents the run time of the method, and does not take into account this (time consuming) process of manipulating the data.

Another feature to note about the cross-validated elastic nets method is that it is (intentionally) a sparse method. Thus, scores are only generated for a small number of genes in each cluster - the genes that are specifically deemed “markers” for that cluster. It is not possible to compare the relative utilities of the genes that are not considered markers - each of those genes are given a score of 0. Thus, beyond a certain number of genes, it is not possible to get any more information from the markers selected by the elastic nets method. (You cannot, for example, request a “bad” marker in order to combine it with the information from other “good” markers).

**Logistic regression.** Logistic regression is proposed as a method for marker selection in [31]. Specifically, a regression is performed on each gene using the cluster label as the response variable. This is translated into a *p*-value via a likelihood ratio (comparing to the null model of logistic regression). The scanpy (version 1.3.7) package includes this method, and thus we are able to run it on sparse data. We again have made some updates to the file _rank_genes_groups.py in the scanpy package to fix some slight errors (that are now fixed in the main release); see the data availability disclosure for where to find this edited file.

RANKCORR. The RANKCORR algorithm is introduced in this paper; the precise procedure is discussed full description of RankCorr. It is important to note that the implementation of RANKCORR that we use here has not been fully optimized. Note that the major step (2) of the SELECT algorithm (Algorithm 1) essentially consists of computing the dot product of each column of a data matrix with the cluster labels *τ*. The only other time consuming portion of SELECT is computing the *ℓ*_2_ norm of a vector. These types of linear algebraic computation have fast implementations that are accessible from Python (e.g. numba). We have not yet optimized the method to take advantage of all possible speed ups since RANKCORR runs quickly enough in our trials.

**Random marker selection.** As a sanity check, we select markers by choosing genes uniformly at random (the same number of markers for each cluster). All of the other methods presented in this work outperform random marker selection by significant margins. For the sake of clarity, the performance of random marker selection is relegated to Additional file 1 (Figures 2–12).

**Seurat** The commonly-used Seurat data analysis package [6] contains implementations of several methods that we consider here, including the Wilcoxon method, the t-test, logistic regression, and MAST. The default method for selecting markers in Seurat is the Wilcoxon method combined with some gene pre-filtering that is designed to speed up the computations. Potentially, the Seurat method could perform better than the standard Wilcoxon rank-sum test (considered in the manuscript) if the researcher is careful to manually tune the different gene filtering thresholds to eliminate uninteresting genes that have high Wilcoxon scores. The Seurat documentation warns that this type of filtering may also eliminate marker genes with weaker differential expression signals, however. We thus do not examine tuning these parameters in this manuscript. See [6] or the Seurat website (https://satijalab.org/seurat/) for further information.

**SCDE.** The SCDE package (we examined version 2.6.0) is implemented in R. Our testing found that it was too slow to be used on real scRNA-seq data sets: it was taking approximately one minute per cell to fit the model (on one core). Since we are performing 5-fold cross-validation, we would need to fit the model approximately 5 times. On one of the smaller data sets (PAUL or ZEISEL), this would require approximately 250 hours of computer time; it would be infeasible to train on the larger data sets. Since we are specifically developing methods for use with the large data sets that are appearing more often, we have excluded SCDE from our final analysis.

**D**^**3**^**E.** D^3^E is also implemented in Python (version 2.7), but it has no support for sparse data structures; thus, running on the 10XMOUSE data set would require a very large amount of memory. Although the method allows for splitting the data into smaller segments (to allow for parallel computation), the full data set needs to be loaded into memory when initializing the process. In addition, when running on the PAUL data set using the faster method-of-moments mode, D^3^E took about 25 minutes running on 10 cores (about 4 hours and 10 minutes total processor time) to find markers for one cluster (vs the rest of the population). Since we need the *p*-values for all (19) clusters for all 5 folds, this method would require approximately 40 hours on 10 cores. Although this is faster than SCDE, this would still be too slow to run on the larger data sets, and thus we exclude D^3^E from our final analysis as well. All of our testing was carried out using the D^3^E source on GitHub (commit efe21d1).

**COMET.** The COMET method [17] is a (streamlined) brute force approach for examining the predictive power of “gene panels” (sets containing up to four genes). COMET inherently has different goals than the majority of the marker selection methods discussed here, and it can not (currently) select more than four genes for a given cell type. Restricting to a very small number of selected markers is useful when the cell types are well-known and a researcher wishes to perform further experimental analysis; COMET is indeed used to guide FACS sorting in [17]. The brute force computational costs do not make COMET a useful tool for data exploration or for providing feedback towards the veracity of a potential clustering, however. In fact, when tested on the PAUL data set, the COMET method took an average of eight minutes to rank pairs of markers for a fixed cluster (resulting in around 2.5 hours of total runtime for all 19 clusters; this would be approximately 12.5 hours to run on all five folds). In addition to this, as of this writing, COMET requires all data to be input as text files or 10x expression files - these are not (especially) sparse formats, and this further limits the data sets that can be studied with this tool (for example, the 10x data files for the full ZHENG dataset require around 600MB of disk space, while the sparse version requires about 100 MB). We thus do not include the full COMET method in our comparisons in this manuscript.

As mentioned in the Background section, however, COMET is based on a statistical test (the XL-mHG test, see [44]) that has some desirable properties for scRNA-seq data. In addition to considering combinations of genes, COMET also ranks individual genes by the average of their XL-mHG *p*-values and (the logarithm of) their fold-changes. These ranks (or the related *p*-values) could be used in a similar fashion to the other differential expression methods considered in this manuscript to select markers for the data sets (e.g. select the top five genes for each cluster). We have elected not to examine this method, however, since we are working with several other differential expression methods based on statistical tests (e.g. Wilcoxon, the t-test).

**scGeneFit.** The scGeneFit method is introduced in the preprint [20]. As mentioned in the Background section, this method attempts to discover markers that are informative about a clustering as a whole rather than determining markers for individual cell types. Specifically, it finds a set of genes *M* such that the projection of the data onto *M* is “optimal.” In this case, optimality means that the distance (after projecting) between different clusters is larger than an input parameter (as much as possible). This optimal projection is defined by a linear program: the variables correspond to genes, and the constraints enforce separation between clusters (there is one constraint for each pair of cells in different clusters). For the sake of efficiency, the current implementation of scGeneFit (https://github.com/solevillar/scGeneFit-python, commit 32dd6a1) considers a random subsample of the constraints; thus, scGeneFit can be applied to data sets of arbitrary size if selecting lower quality markers is acceptable. The trade-offs between efficiency and marker quality are not yet fully explored in the preprint [20], however (for example, the number of constraints required for quality should probably be related to the number of clusters; this method of random sampling also seems to deemphasize rare cell types).

We ran scGeneFit using parameters suggested on the scGeneFit GitHub page and altering the number of constraints so that the method ran in a reasonable amount of time on the PAUL and ZEISEL data sets (approximately 10 minutes to run on one fold). The results that we obtained were somewhat suboptimal, however, especially on the PAUL data set. Additionally, the supercomputer architecture used for our analysis changed before we collected data using scGeneFit; thus, comparisons of the timing of scGeneFit to the other methods would not be valid. Therefore, further considering that scGeneFit [20] is presented in a preprint (and is thus subject to significant future change), we report our scGeneFit results in Additional file 1 (Figures 2 and 3 for ZEISEL; Figures 5 and 6 for PAUL) rather than the main manuscript.

### Generating marker sets of different sizes from algorithms other than RANKCORR

For a fixed data set, we need to select markers for a given clustering - not just markers for a single cluster. Here we describe how we select a specific number of markers and how we merge lists of markers for individual clusters to make a marker list for the entire clustering.

For a differential expression method, we proceed in a one-vs-all fashion: letting *C* denote the number of clusters in the given clustering, we use the differential expression methods to find *C* vectors of *p*-values; the *i*-th vector corresponds to the comparison between cluster *i* and all of the other cells. For the sake of simplicity, we then include an equal number of markers for each cluster to create a set of markers for the clustering.

For example, we consider the classification error rate when the marker list consists of the three genes with the smallest *p*-values from each cluster (with duplicates removed). As previously mentioned, this is a vast oversimplification of a tough problem - how to merge these lists of *p*-values in an optimal way, making sure that we have good representation of each cluster - but it allows for us to quickly and easily compare the methods that we present here. (Note that we would probably want to choose more markers for a cluster in which all *p*-values were large - we probably need more coordinates to distinguish this cluster from all of the others, even if those coordinates are not particularly informative. Thus, setting a *p*-value threshold could potentially perform worse than the method outlined here, as we may not select any markers for a certain cluster with a thresholding method.) The process of merging lists of *p*-values is left for future work.

For the elastic nets method, which selects one list of markers as optimal (without giving a score for all of the markers), we apply a similar strategy to approximate selecting a small number of markers. In particular, we choose an equal number of markers with the highest score for each cluster until we run out of markers to select. For example, when attempting to select 20 markers per cluster, we may include the top 20 markers for one cluster and all 18 of the markers that are selected for a different cluster.

### Ranking the performance of the methods in Fig. 2

The numbers in the cells of the tables in Fig. 2 are meant to provide an approximate ranking of the marker selection methods as different numbers of markers are selected. The supervised classification metrics are ranked separately from the unsupervised clustering metrics; that is, each column in a table in Fig. 2 contains *two* rankings of the marker selection methods. A rank of 1 corresponds to the best method; larger rank values indicate worse performance.

Each rank is meant to capture the results of multiple evaluation metrics when the methods select a range of markers (six metrics for the classification table cells and three metrics for the clustering table cells - see Table 3; the ranges of numbers of markers appear in the first row of each table in Fig. 2). We thus first calculate a score summarizing the metrics in a range of selected markers. Here, will call these the AoM scores, so called because they are based on an Average of Medians. Briefly, for a fixed method and a fixed range of markers, the AoM score is computed as an average across the relevant evaluation metrics. Each quantity in the average is the median (computed over the fixed range of markers) of the performance of the method according to one of the relevant evaluation metrics. That is, for a fixed method and a fixed range [*a*,*b*] of markers, we define

11 $$ \text{AoM} = \text{Average}_{m\in \text{metrics}}\left\{ \text{median}_{[a,b]}\{ m(\text{method})\} \right\}  $$

Some considerations about the definitions of the AoM scores is discussed in more detail in the following paragraphs. The ranks are then determined from the relative AoM scores of the different methods.

We calculate the AoM scores from the data that appear in Figs. 4, 5, 6, 7, 8, 9 and 10 without any extrapolation (that is, the curves themselves are not considered in these calculations, only the points). For this reason, the AoM scores are quite sensitive to the marker ranges (the bins in the top row of the tables in Fig. 2): since the performance of the methods improve as more markers are selected, methods that have a data point towards the right edge of one of the marker ranges will generally be rated more favorably by summary calculations. This effect will be especially pronounced when analyzing the leftmost portions of Figs. 4, 5, 6, 7, 8, 9 and 10 (that is, when considering small sets of markers), where the curves are generally improving rapidly. To partially account for this issue, the AoM scores are based on the median performance value produced by the methods in a range of markers.

We choose to average these medians as a way to summarize them in our computation of the AoM scores. This is mainly due to the fact that we want each evaluation metric to contribute equally to the summary score. For example, for a fixed method, if one evaluation metric produces poor results while the other metrics are okay, we still want to include information about this outlier in our AoM score. As discussed previously, the evaluation metrics all produce different ways of examining the information contained in a set of markers; it is thus a warning sign if even one metric is poor. The evaluation metrics are on the same scale (all produce values from 0 to 1, with larger values indicating better performance), so standardization is not needed.

We report ranks in Fig. 2 (rather than the AoM scores) since the values of these AoM scores are relatively uninformative and difficult to read (for example, a difference of 0.02 between two AoM scores is quite large - it represents a 2% difference between the two methods). The actual AoM values can be found in the data in the GitHub repository related to this paper (https://github.com/ahsv/marker-selection-code).

### Generating synthetic data based on scRNA-seq data

In order to generate synthetic data that is made to look like an experimental droplet-based scRNA-seq data set, we use the Splat method from the R Splatter package (version 1.6.1) [26] in R version 3.5.0.

We use the data set consisting of purified (CD19+) B cells from [2] in order to estimate the Splat simulation parameters. In [2], the authors analyzed this dataset and saw only one cluster, suggesting that it consists mostly of one cell type. We have also combined it with the full ZHENGFULL dataset from [2] (see the descriptions of the experimental data sets) and observed good overlap with the cluster that the authors identified as B cells in ZHENGFULL when looking at a two dimensional UMAP visualization. This overlap appears in Additional file 1, Figure 18.

Testing with Splatter showed that including dropout in the Splat simulation resulted in a simulated data set with a higher fraction of entries that are 0 than the original dataset. On the other hand, not including dropout resulted in similar fractions of entries that are 0 in the simulated and original datasets. Taking into account the fact that the Splat dropout randomly sets entries to 0 regardless of the size of those entries (a practice that we would argue is an unrealistic representation of actual dropout), we do not include additional dropout in our Splat simulations.

In the Splat method, differential expression is simulated by generating a multiplicative factor for each gene that is applied to the gene mean before cell counts are created - a factor of 1 means that the gene is not differentially expressed. These multiplicative factors come from a lognormal distribution with location 0.1 and scale 0.4 - the default values in the Splatter package. We have not attempted to tweak these default parameters in this work. Using the default parameters, many of the “differentially expressed” genes have a differential expression multiplier that is between 0.9 and 1.1; for these genes, the gene mean is barely different between the two clusters. This creates a significant number of differentially expressed genes that are difficult to detect.

In our synthetic data sets, we ask for Spatter to simulate two groups: 10% of the genes in the first group are differentially expressed (i.e. have a differential expression multiplier not equal to 1) and none of the genes in the second group are differentially expressed. In this way, all differentially expressed genes can be considered to be marker genes for the first group - there are no overlaps between markers for the first and second groups. The direction of differential expression is randomly determined for each gene. Since the differentially expressed genes are chosen at random, this means that many of the genes that are labeled as differentially expressed in the output data show low expression levels (often they are expressed in less than 10 cells).

As discussed in the simulated data results, we create 20 different simulated data sets from the CD19+ B cells dataset. See Fig. 11 for a diagramme of the set-up. For all 20 simulated data sets, we simulate 5000 cells and the same number of genes that we input. The first 10 data sets are created by using the full (unfiltered) information from 10 random samples of 5000 cells from the B cell data set. This procedure results in synthetic data sets that consist of 5000 cells and about 12000 genes (the number of nonzero genes depends on the subsample). We label our results on these data sets under the heading “all genes used for simulation.”

To attempt to mitigate the issue of extremely similar gene means between the two clusters in some of the “differentially expressed” genes, we filter the genes of the simulated data via the method introduced in [1]: namely, place the genes in 20 bins based on their mean expression levels and select the genes with the highest dispersion from each bin. Using this method, we select the top 5000 most variable genes from the simulated data and we then use only these genes for marker selection. In the figures, we report these data under the heading “filtering after simulation.”

This type of gene filtering is also common in the literature, and we thought the affect of filtering on marker selection deserved further consideration. Thus, the second 10 simulated data sets are created by using only the top 5000 most variable genes in the original data as the input to Splatter. In this way, we are forcing the differentially expressed genes to look like genes that were originally highly variable. The results on these data are labeled “filtering before simulation.”

We again use the cell_ranger flavor of the filter_genes_dispersion function in the scanpy Python package for all variable gene selection. Occasionally, this results in only 4999 genes selected; in those cases, we consider 4999 genes (rather than 5000) in the filtered data sets. See the scripts and notebooks on our GitHub repository (link in the data availability statement) for precise information about when this occurred.

## Supplementary information

**Additional file 1** Supplementary figures. This file (available in pdf format) contains figures that are supplementary to the data presented in this manuscript. These figures include:
Plots of all supervised clustering metrics (see Table 1) for all methods (including edgeRdet, MASTdet, and random marker selection when the relevant data was collected) on all four experimental data sets generated using both the NCC and RFC.
Plots of the unsupervised clustering metrics (see Table 1) for all methods (including edgeRdet, MASTdet, and random marker selection when the relevant data was collected) on the Zeisel, Paul, and ZhengFilt data sets.
A visualization of the variance in the classification error rate when using the random forests classifier (RFC).
Plots of the data that were used to choose the value of *k* (see the discussion on the selection of Louvain parameters) that was used to compute the unsupervised clustering metrics on the Zeisel and Paul data sets.
A comparison of UMAP plots of the ZhengFull data set when labeled by (a) the biologically motivated bulk labels that were used as the “ground truth” cell types for marker selection in this manuscript, and (b) a Louvain clustering that was generated for this work. The Louvain clustering in (b) was used to guide the selection of *k* (see the discussion on the selection of Louvain parameters) to compute the unsupervised clustering metrics on the ZhengFilt data set.
A UMAP plot of the purified CD19+ B cell data set that was used to generate the Simulated data illuminates the precise performance characteristics of marker selection methods in this work combined with the ZhengFull data set.

## Data Availability

The experimental data sets analysed during the current study are publicly available. They can be found in the following locations: • Zeisel is found on the website of the authors of [24]: http://linnarssonlab.org/cortex/. The data are also available on the GEO (GSE60361). • Paul is found in the scanpy Python package - we consider the version obtained by calling the scanpy.api.datasets.paul15() function. The clustering is included in the resulting Anndata object under the heading paul15_clusters. The data are also available on the GEO (GSE72857). • ZhengFull and ZhengFilt are (subsets) of the data sets introduced in [2]. The full data set can be found on the 10x website (https://support.10xgenomics.com/single-cell-gene-expression/datasets/1.1.0/fresh_68k_pbmc_donor_a) as well as on the SRA (SRP073767). The biologically motivated bulk labels can be found on the scanpy_usage GitHub repository at https://github.com/theislab/scanpy_usage/blob/master/170503_zheng17/data/zheng17_bulk_lables.txt(we use commit 54607f0). • 10xMouse is available for download on the 10x website (https://support.10xgenomics.com/single-cell-gene-expression/datasets/1.3.0/1M_neurons). The clustering analysed in this manuscript can be found on the scanpy_usage GitHub repository (https://github.com/theislab/scanpy_usage/tree/master/170522_visualizing_one_million_cells; we consider commit ba6eb85) The synthetic data analysed in this manuscript is based on the CD19+ B cell data set from [2]. This B cell data set can be found on the 10x website at https://support.10xgenomics.com/single-cell-gene-expression/datasets/1.1.0/b_cells. The synthetic data sets themselves are available from the author on request. All scripts that were used for marker selection and data processing (including implementations of Spa and RankCorr) can be found at the GitHub repository located at https://github.com/ahsv/marker-selection-code. These scripts also include Jupyter notebooks that produce interactive versions of the figures in this manuscript (allowing for the user to zoom in, remove some of the curves, and more). A streamlined implementation of RankCorr (with documentation) can additionally be found at https://github.com/ahsv/RankCorr.

## Abbreviations

scRNA-seq: Single-cell RNA sequencing
UMI: Unique molecular identifier
FACS: Fluorescence-activated cell sorting
PCA: Principal component analysis (for dimensionality reduction)
UMAP: Uniform manifold approximation and projection (for dimensionality reduction)
Log. Reg: Logistic regression. *Marker selection evaluation measures*
NCC: Nearest centroids classifier
RFC: Random forest classifier
MCC: Matthews correlation coefficient
ARI: Adjusted Rand index
AMI: Adjusted mutual information
FMS: Fowlkes-Mallows score
TPR: True positive rate
FPR: False positive rate
ROC curve: Receiver operating characteristic curve
AoM: Average of medians

## Footnotes

1 Software is available for download at https://github.com/ahsv/RankCorr.

2 Recall that *X*_*j*_ represents the counts of gene *j* across the entire population of cells. Since the genes are ranked separately, a count of 0 will be given a different rank in each gene.

3 It may be possible to filter so as to retain only the interesting genes while reducing the size of the data set (this is the goal, for example, of the different filtering and preprocessing schemes for marker selection in the Seurat package; see the description of Seurat in the marker selection algorithms); it is important to carefully consider the effects of these preprocessing choices on the final results.

4 In the usual case with noisy experimental data, *v* has a unique largest entry, and thus $\tilde {v}$ will have one nonzero entry so that it is pointing along one of the coordinate axes.
