# Supplementary material for: A rank-based marker selection method for high throughput scRNA-seq data
Source: BMC Bioinformatics. 2020 Oct 23;21:477. doi: 10.1186/s12859-020-03641-z (PMC7585212; doi:10.1186/s12859-020-03641-z)
Supplement: Supplementary file 1 — Additional file 1 Supplementary figures. This file (available in pdf format) contains figures that are supplementary to the data presented in this manuscript. These figures include: Plots of all supervised clustering metrics (see Table 1) for all methods (including edgeRdet, MASTdet, and random marker selection when the relevant data was collected) on all four experimental data sets generated using both the NCC and RFC. Plots of the unsupervised clustering metrics (see Table 1) for all methods (including edgeRdet, MASTdet, and random marker selection when the relevant data was collected) on the Zeisel, Paul, and ZhengFilt data sets. A visualization of the variance in the classification error rate when using the random forests classifier (RFC). Plots of the data that were used to choose the value of k (see the discussion on the selection of Louvain parameters) that was used to compute the unsupervised clustering metrics on the Zeisel and Paul data sets. A comparison of UMAP plots of the ZhengFull data set when labeled by (a) the biologically motivated bulk labels that were used as the “ground truth” cell types for marker selection in this manuscript, and (b) a Louvain clustering that was generated for this work. The Louvain clustering in (b) was used to guide the selection of k (see the discussion on the selection of Louvain parameters) to compute the unsupervised clustering metrics on the ZhengFilt data set. A UMAP plot of the purified CD19+ B cell data set that was used to generate the Simulated data illuminates the precise performance characteristics of marker selection methods in this work combined with the ZhengFull data set. [file 12859_2020_3641_MOESM1_ESM.pdf]

A rank-based marker selection method for high throughput scRNA-seq data: Additional file 1, supplementary figures

Anna C. Gilbert

Alexander Vargo

June 20, 2020

This file contains figures to supplement the content in the main manuscript.

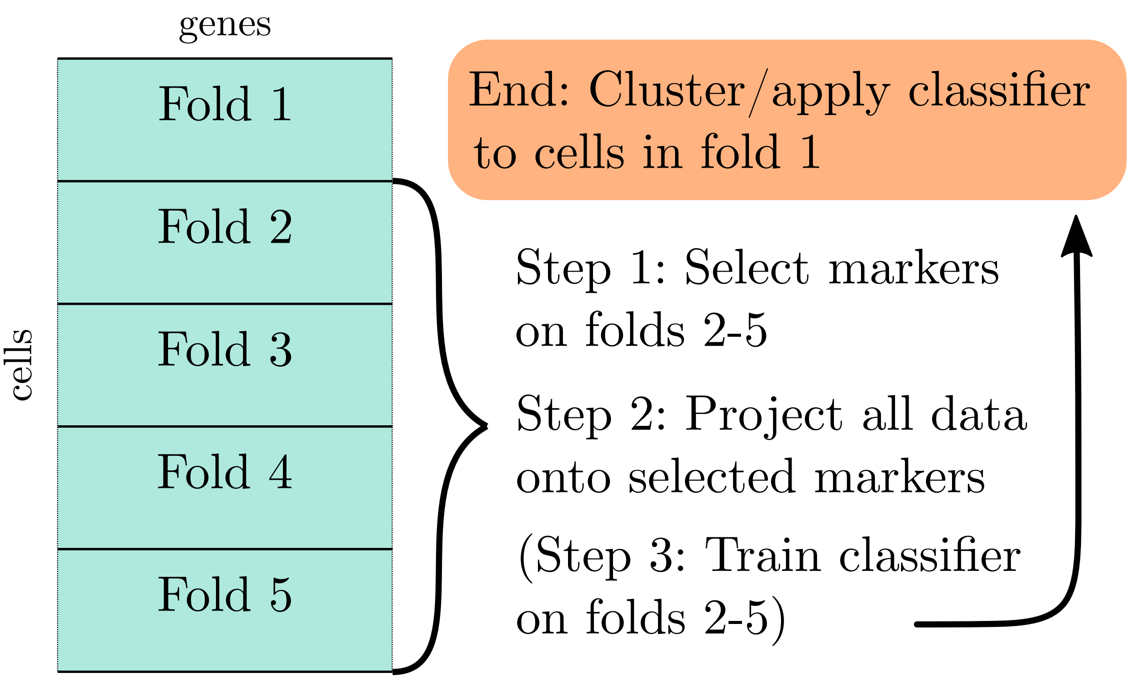

Figure 1: A visual description of 5 fold cross-validation

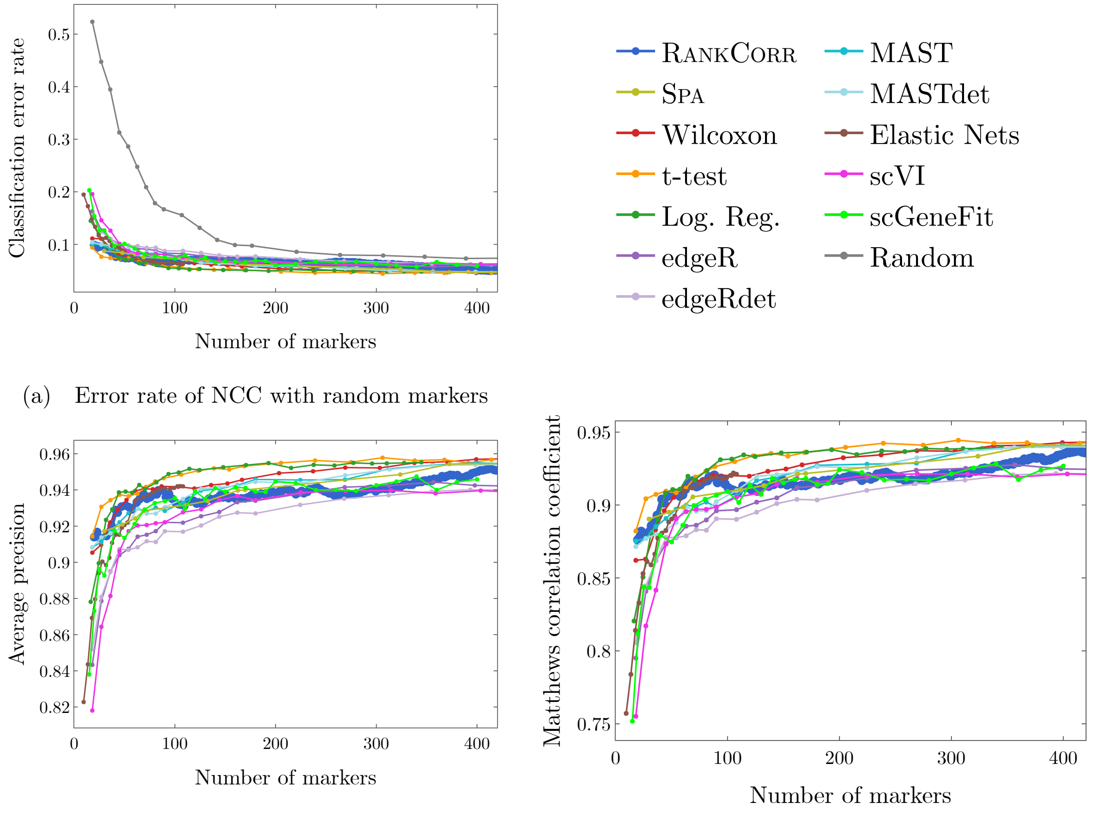

(a) Error rate of NCC with random markers

(b) Average precision of NCC

(c) Matthews correlation coefficient of NCC

Figure 2: Supervised classification metrics for the Zeisel data set using the nearest centroid classifier (NCC). Included is the performance of the scGeneFit method. (a) is the classification error rate including a curve corresponding to random marker selection (the curve that starts at around 50% error). (b) contains the precision and (c) contains the Matthews correlation coefficient. Data from random marker selection are not included in figures (b) and (c) for clarity. Note that the curves in (b) are similar in shape to the curves in (c); they are also similar in shape to the classification accuracy (1− classification error rate from Figure 4(a) of the main manuscript).

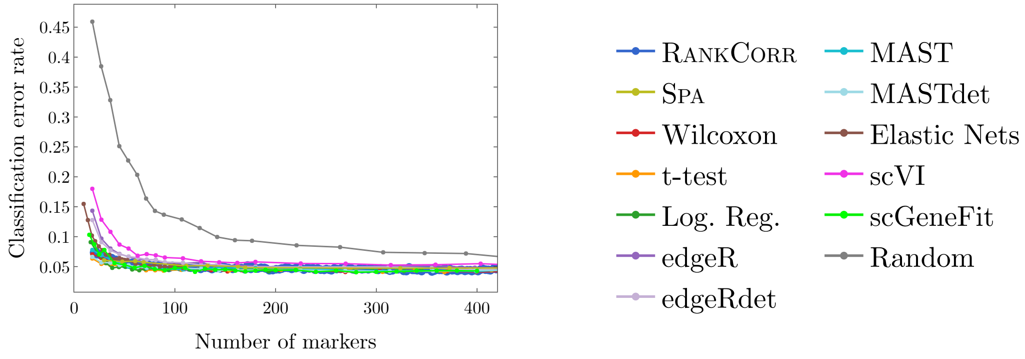

(a) Error rate of RFC with random markers

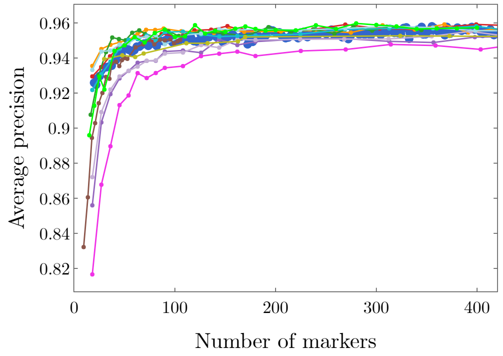

(b) Average precision of RFC

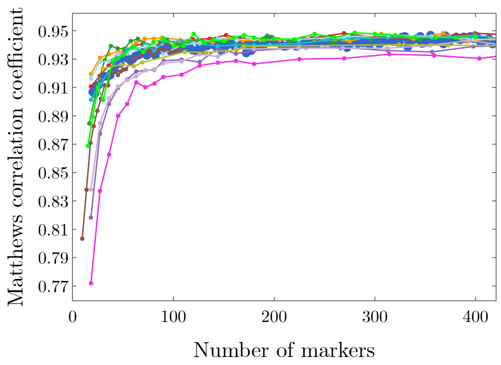

(c) Matthews correlation coefficient of RFC

Figure 3: Supervised classification metrics for the Zeisel data set using the random forests classifier (RFC). Included is the performance of the scGeneFit method. (a) is the classification error rate including a curve corresponding to random marker selection (the curve that starts at around 45% error). (b) contains the precision and (c) contains the Matthews correlation coefficient. Data from random marker selection are not included in figures (b) and (c) for clarity. Note that the curves in (b) are similar in shape to the curves in (c); they are also similar in shape to the classification accuracy (1− classification error rate from Figure 4(c) of the main manuscript).

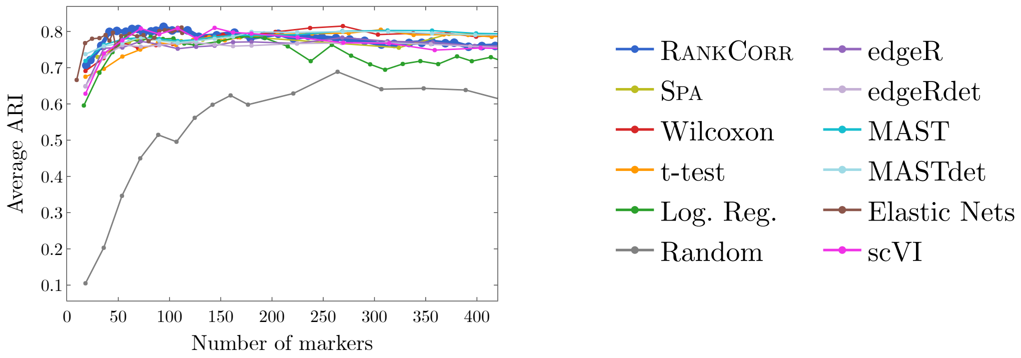

(a) Average ARI scores

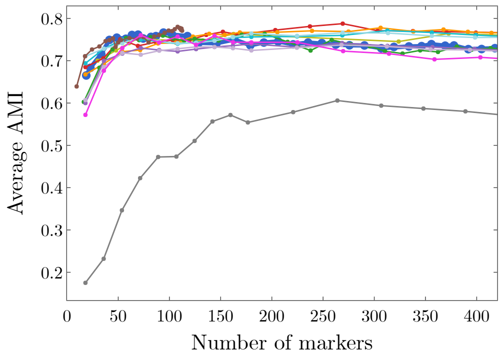

(b) Average AMI scores

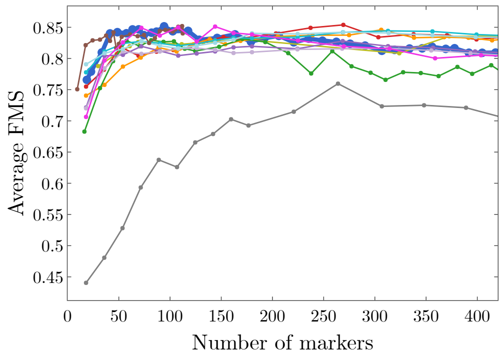

(c) Average Fowlkes-Mallows scores

Figure 4: Unsupervised clustering metrics for the Zeisel data set including data from random marker selection. The curve corresponding to random marker selection is shown in grey and is the lowest (worst) curve in all three plots. The ARI score is shown in (a), the AMI score is shown in (b), and the Fowlkes-Mallows score is shown in (c). The clustering is carried out using 5-fold cross validation and scores are averaged across folds.

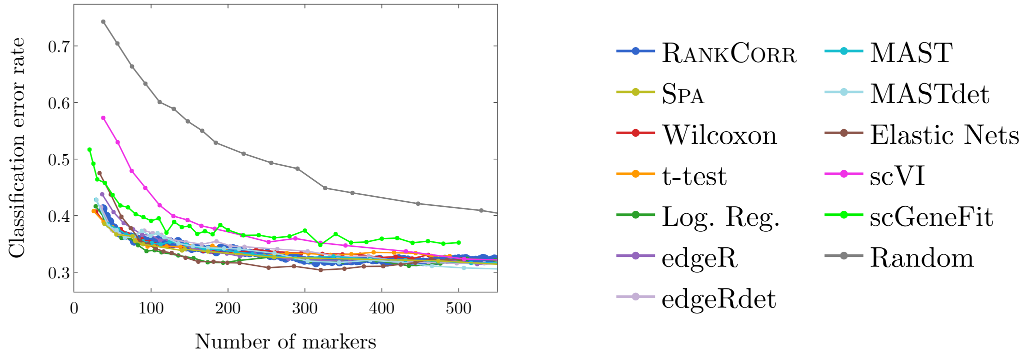

(a) Error rate of NCC with random markers

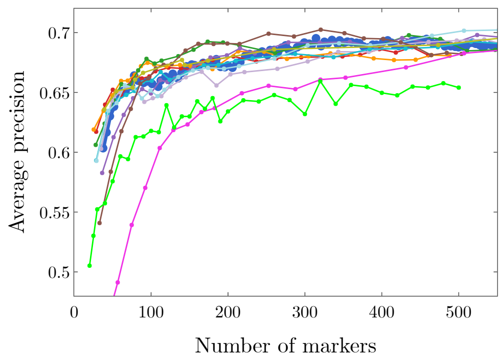

(b) Precision of NCC

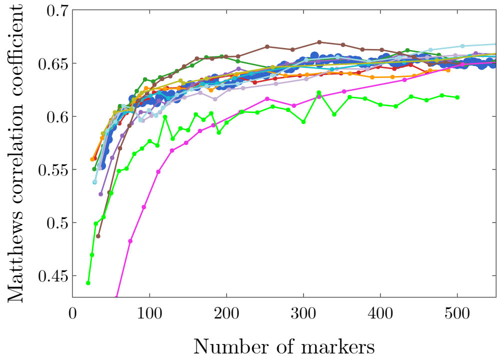

(c) Matthews correlation coefficient of NCC

Figure 5: Supervised classification metrics for the Paul data set using the nearest centroid classifier (NCC). Included is the performance of the scGeneFit method. (a) is the classification error rate including a curve corresponding to random marker selection (the curve that starts at around 75% error). (b) contains the precision and (c) contains the Matthews correlation coefficient. Data from random marker selection are not included in figures (b) and (c) for clarity. Note that the curves in (b) are similar in shape to the curves in (c); they are also similar in shape to the classification accuracy (1− classification error rate from Figure 6(a) of the main manuscript).

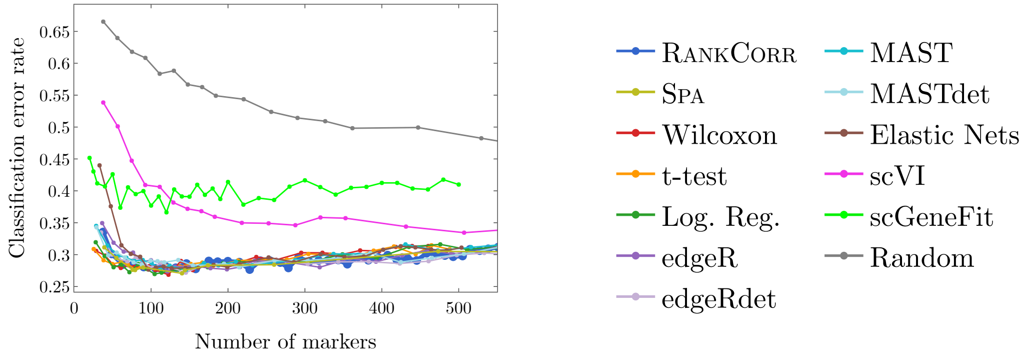

(a) Error rate of RFC with random markers

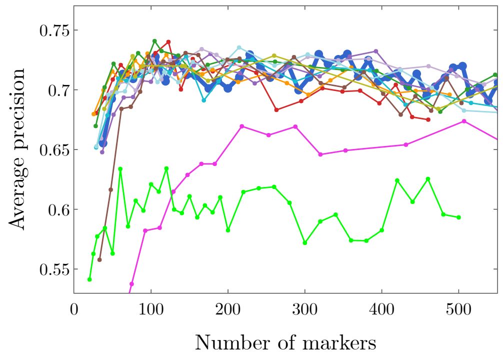

(b) Precision of RFC

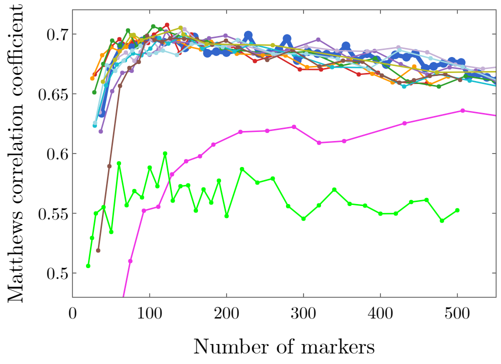

(c) Matthews correlation coefficient of RFC

Figure 6: Supervised classification metrics for the Paul data set using the random forests classifier (RFC). Included is the performance of the scGeneFit method. (a) is the classification error rate including a curve corresponding to random marker selection (the curve that starts at around 65% error). (b) contains the precision and (c) contains the Matthews correlation coefficient. Data from random marker selection are not included in figures (b) and (c) for clarity. Note that the curves in (b) are similar in shape to the curves in (c); they also similar in shape to the classification accuracy (1− classification error rate from Figure 6(c) of the main manuscript).

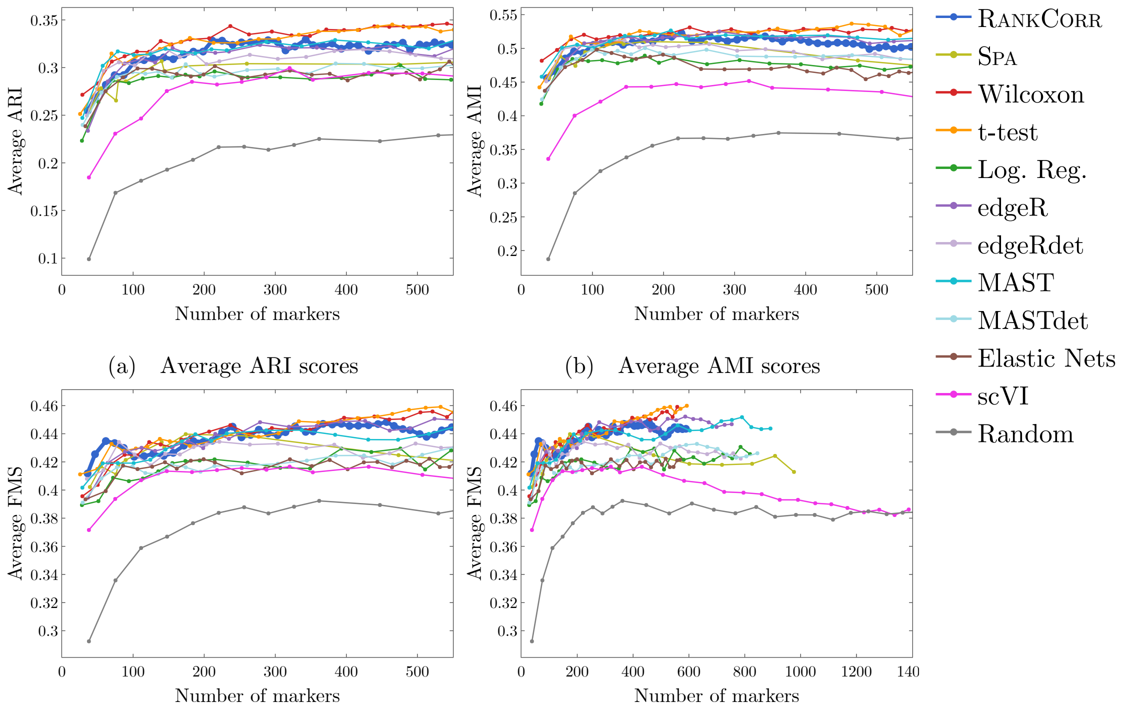

(a) Average ARI scores

(b) Average AMI scores

(c) Average Fowlkes-Mallows scores

(d) Tail behavior of Fowlkes-Mallows scores

Figure 7: Unsupervised clustering metrics for the Paul data set including data from random marker selection. The curve corresponding to random marker selection is shown in grey and is the lowest (worst) curve in all four plots. The ARI score is shown in (a), the AMI score is shown in (b), and the Fowlkes-Mallows score is shown in (c). (d) contains the FM scores for larger numbers of markers selected, showing the scVI does approach the behavior of random marker selection in this case. Each clustering is carried out using 5-fold cross validation and scores are averaged across folds.

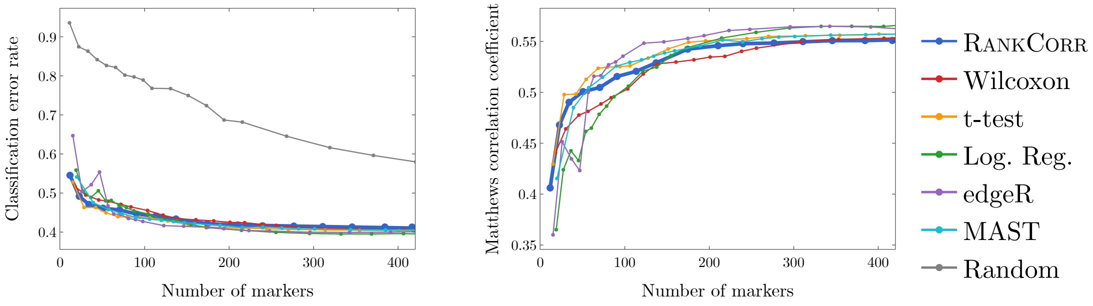

(a) Error rate of NCC with random markers

(b) Matthews correlation coefficient of NCC

Figure 8: Supervised classification metrics for the ZhengFilt data set using the nearest centroid classifier (NCC). (a) is the classification error rate including a curve corresponding to random marker selection (the curve that starts at around 90% error). (b) contains the Matthews correlation coefficient. Data from random marker selection are not included in (b). Note that (b) is similar in shape to the classification accuracy (1− classification error rate from Figure 8(a) of the main manuscript).

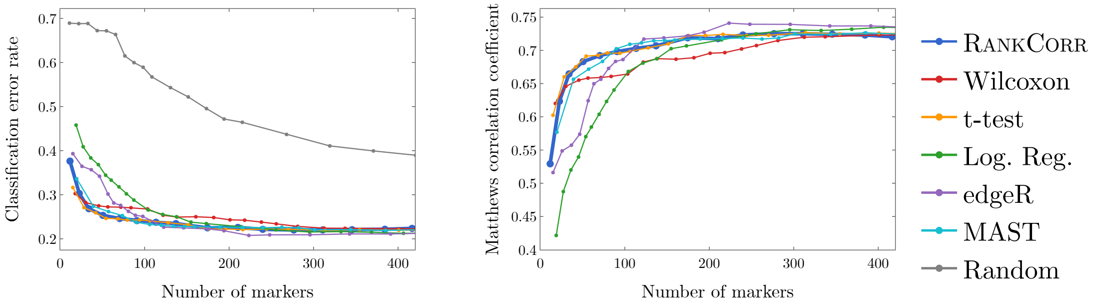

(a) Error rate of RFC with random markers

(b) Matthews correlation coefficient of RFC

Figure 9: Supervised classification metrics for the ZhengFilt data set using the random forests classifier (RFC). (a) is the classification error rate including a curve corresponding to random marker selection (the curve that starts at around 70% error). (b) contains the Matthews correlation coefficient. Data from random marker selection are not included in (b). Note that (b) is similar in shape to the classification accuracy (1− classification error rate from Figure 9(a) of the main manuscript).

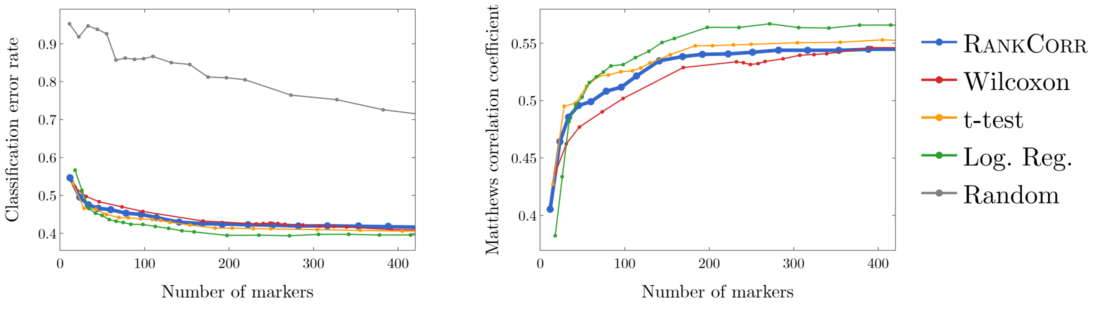

(a) Error rate of NCC with random markers

(b) Matthews correlation coefficient of NCC

Figure 10: Supervised classification metrics for the ZhengFull data set using the nearest centroid classifier (NCC). (a) is the classification error rate including a curve corresponding to random marker selection (the curve that starts at around 90% error). (b) contains the Matthews correlation coefficient. Data from random marker selection are not included in (b). Note that (b) is similar in shape to the classification accuracy (1− classification error rate from Figure 8(c) of the main manuscript).

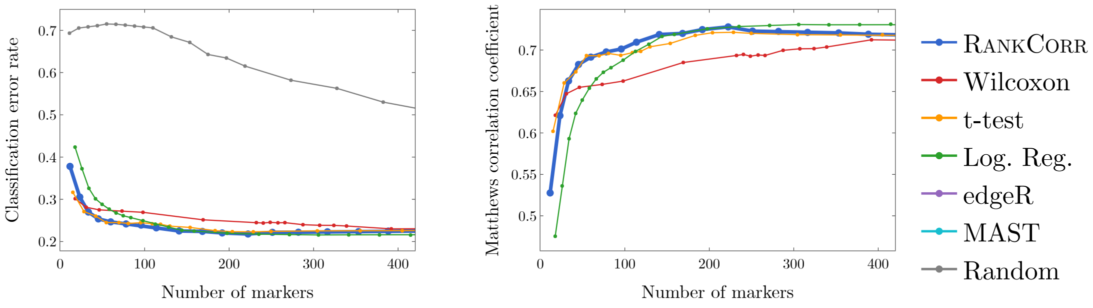

(a) Error rate of RFC with random markers

(b) Matthews correlation coefficient of RFC

Figure 11: Supervised classification metrics for the ZhengFull data set using the random forests classifier (RFC). (a) is the classification error rate including a curve corresponding to random marker selection (the curve that starts at around 70% error). (b) contains the Matthews correlation coefficient. Data from random marker selection are not included in (b). Note that (b) is similar in shape to the classification accuracy (1− classification error rate from Figure 9(c) of the main manuscript).

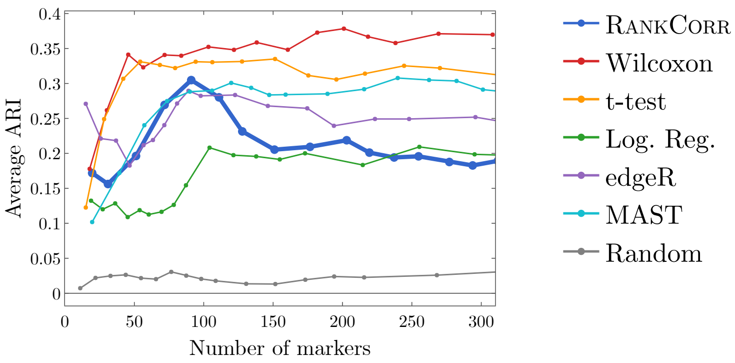

(a) Average ARI scores

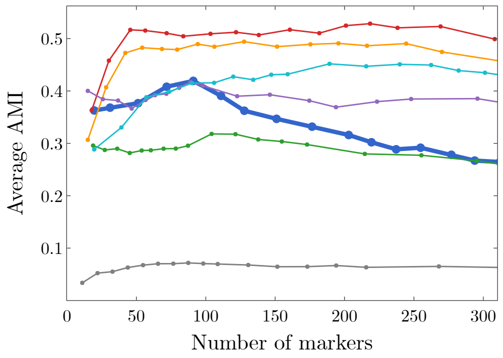

(b) Average AMI scores

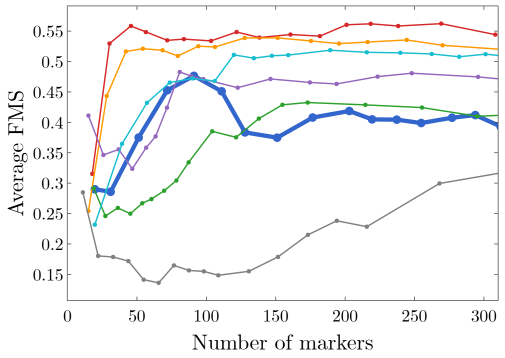

(c) Average Fowlkes-Mallows scores

Figure 12: Unsupervised clustering metrics for the ZhengFilt data set including data from random marker selection. The curve corresponding to random marker selection is shown in grey and is the lowest (worst) curve in all three plots. The ARI score is shown in (a), the AMI score is shown in (b), and the Fowlkes-Mallows score is shown in (c). Each clustering is carried out using 5-fold cross validation and scores are averaged across folds.

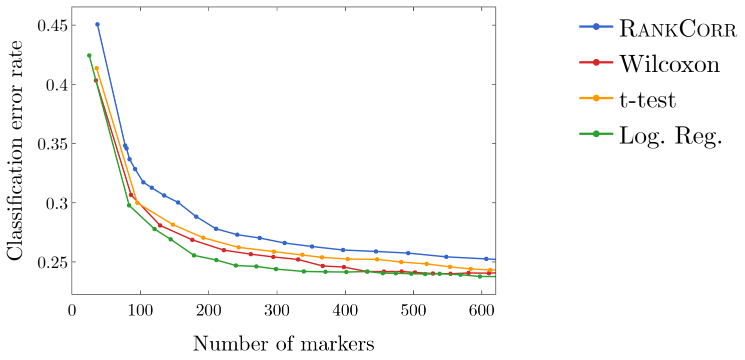

(a) Error rate of NCC

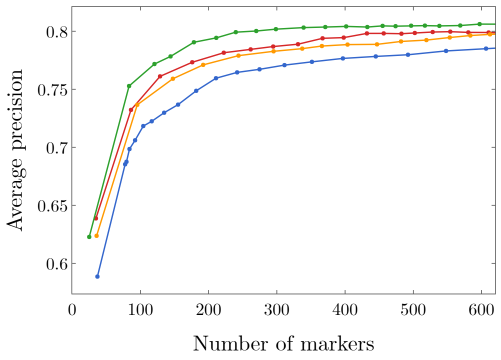

(b) Average precision of RFC

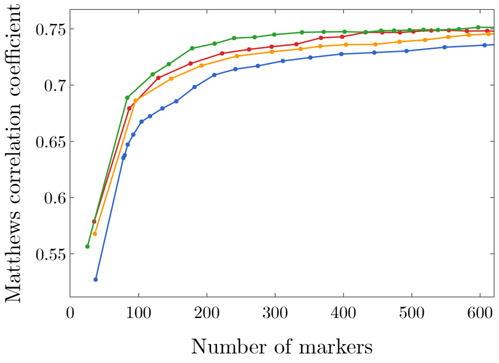

(c) Matthews correlation coefficient of NCC

Figure 13: Supervised classification metrics for the 10xMouse data set using the nearest centroid classifier (NCC). (a) contains the classification error rate curves, (b) contains the average precision curves, and (c) contains the Matthews correlation coefficient curves. We do not compare to random markers on this data set.

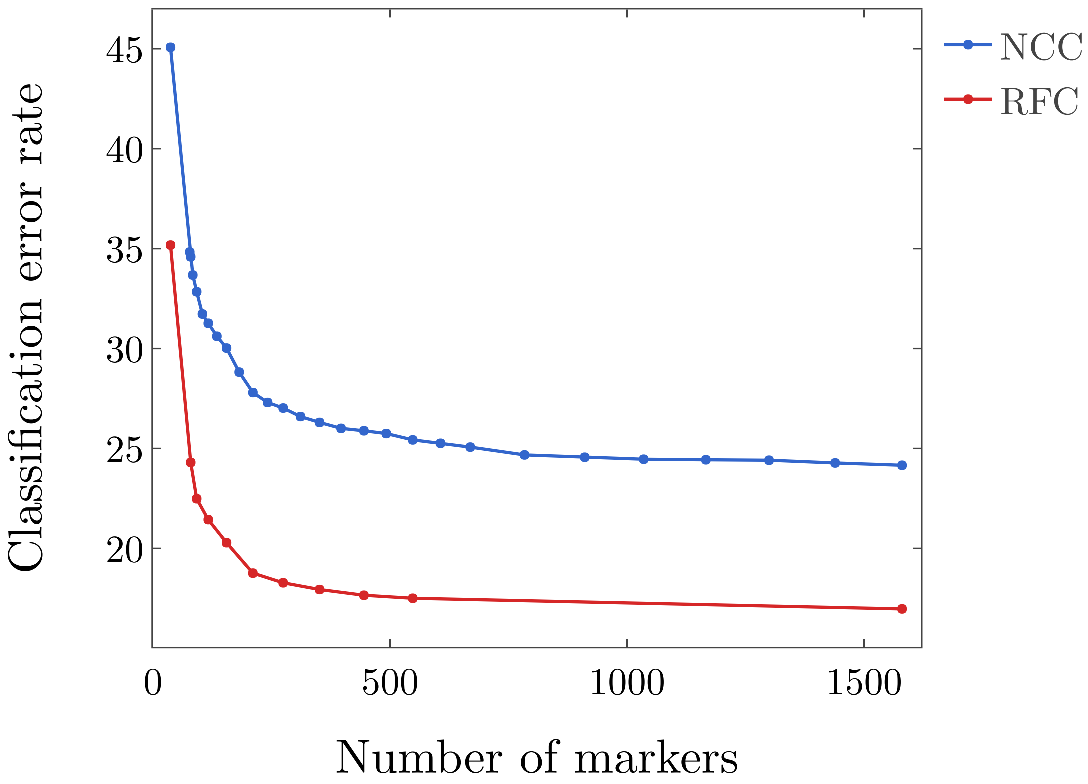

Figure 14: A comparison of the nearest centroid classifier (NCC) and the random forest classifier (RFC) using the RankCorr method on the 10xMouse data set

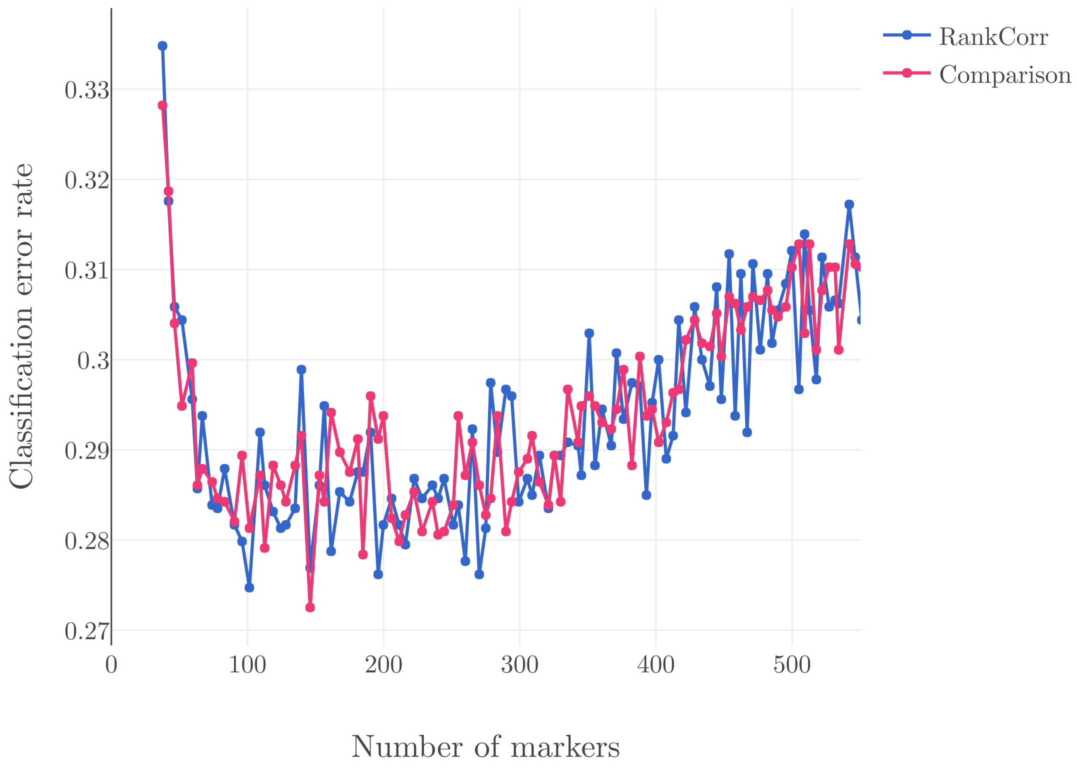

Figure 15: The classification accuracy under the RFC on the PAUL data set (see the Methods in the main manuscript) run twice with the same markers used for each point. Significant variation is observed in the classification accuracy over the two classification attempts. Differences of nearly 2% are observed between the two curves.

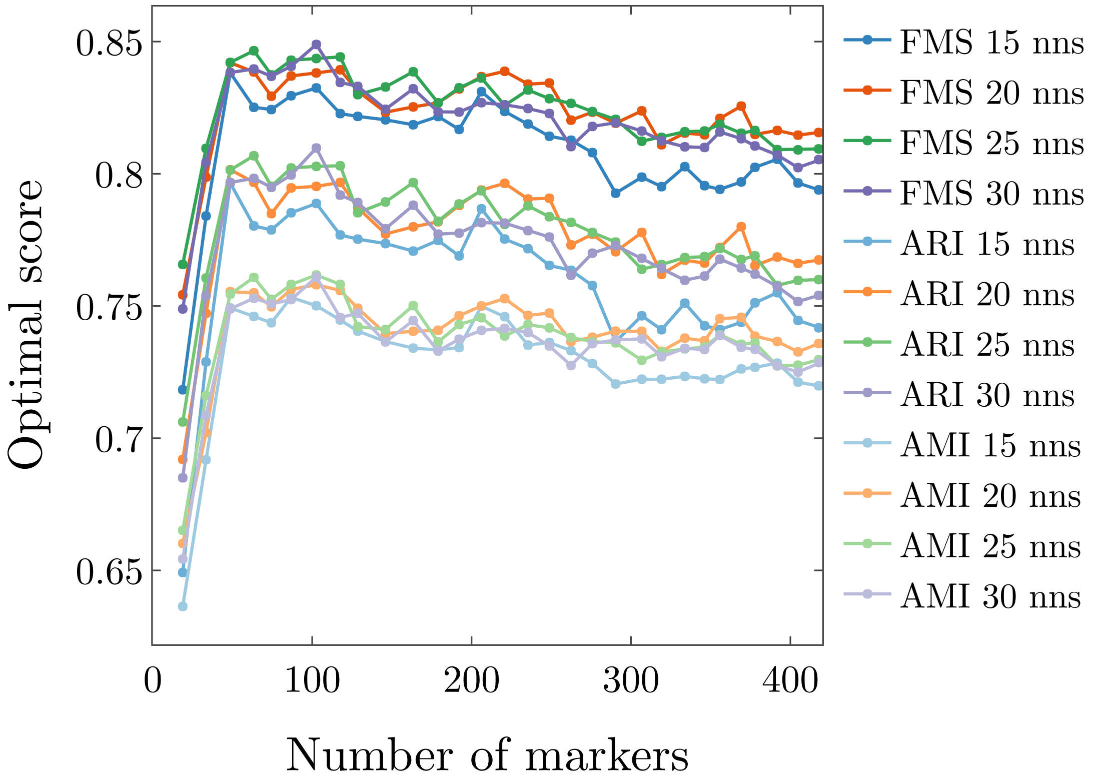

Figure 16: Effect of changing the number of nearest neighbors on the ARI, AMI, and FM scores for the Zeisel data set using RankCorr to select markers. Clustering was performed with Louvain and the scores were optimized over the resolution. It appears that 15 nearest neighbors is too few, while 30 nearest neighbors is too many.

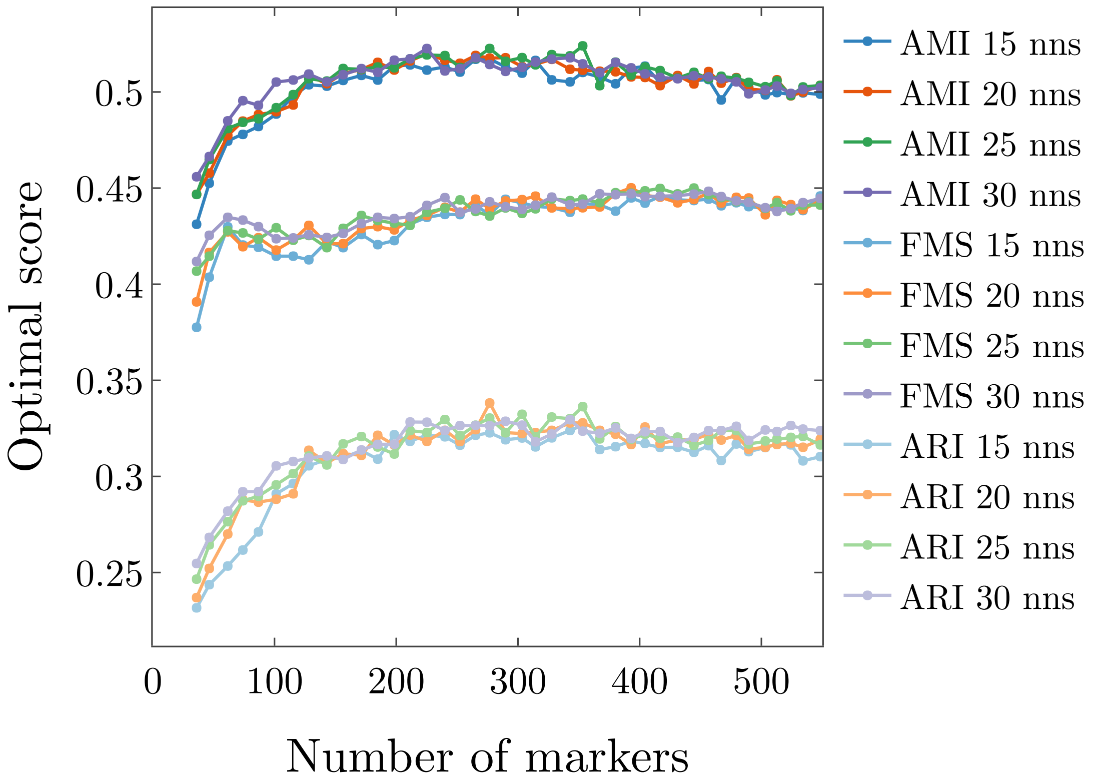

Figure 17: Effect of changing the number of nearest neighbors on the ARI, AMI, and FM scores for the Paul data set using RankCorr to select markers. Clustering was performed with Louvain and the scores were optimized over the resolution. All of the choices of numbers of nearest neighbors produce similar curves for all three scores. Choosing 30 nearest neighbors appears to provide increased performance for small numbers of markers.

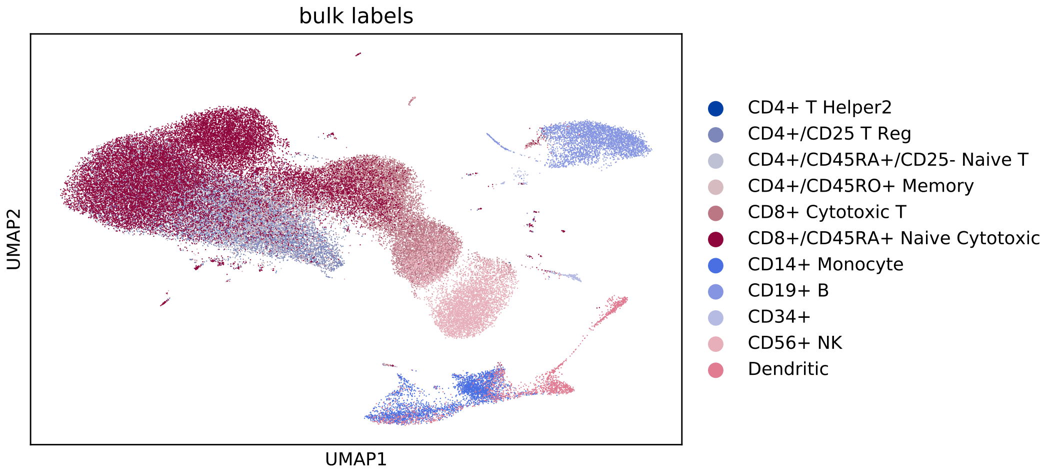

(a)

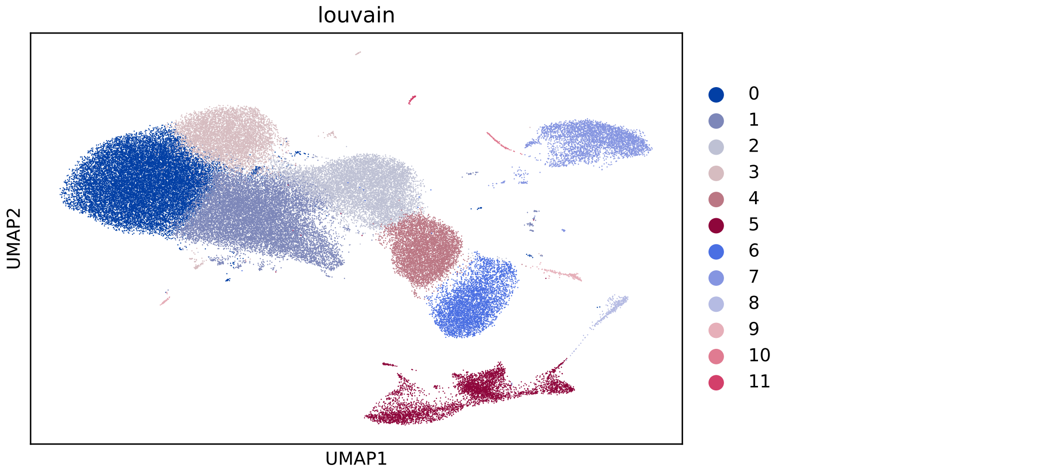

(b)

Figure 18: Clustering the 68k PBMC data set from [1] (reference [2] from the main manuscript) with Louvain clustering. (a) contains a UMAP plot of the bulk labels. (b) is a UMAP plot of a Louvain clustering of the data set. It was created by first filtering to the 1000 most variable genes using the `cell_ranger` flavor of the `filter_genes_dispersion` function in the `scanpy` python package. The Louvain algorithm was run on the top 50 PCs and used 25 nearest neighbours for each cell with a resolution parameter of 0.3. The Louvain clustering solution subjectively looks similar to the bulk labels. The ARI for the clustering compared to the bulk labels is 0.345, the AMI is 0.565, and the FMS is 0.462 (these values have been rounded to 3 significant digits).

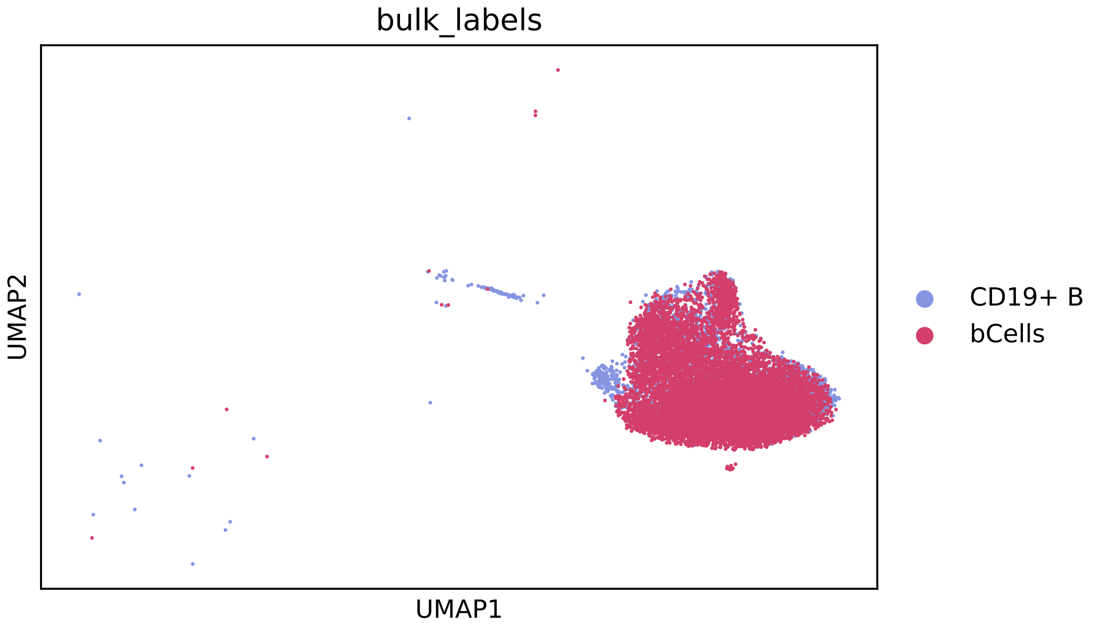

Figure 19: UMAP projection of the data consisting of ZhengFull combined with the isolated CD19+ B cell data set from [1] (reference [2] from the main manuscript) that was used to estimate parameters in Splatter simulations for generating synthetic data. We show only the isolated CD19+ sample (labeled "bCells") and the cluster of B cells from ZhengFull. The overlap between the two clusters is quite good.

References

[1] Zheng, G.X.Y., Terry, J.M., Belgrader, P., Ryvkin, P., Bent, Z.W., Wilson, R., Ziraldo, S.B., Wheeler, T.D., McDermott, G.P., Zhu, J., Gregory, M.T., Shuga, J., Montesclaros, L., Underwood, J.G., Masquelier, D.A., Nishimura, S.Y., Schnall-Levin, M., Wyatt, P.W., Hindson, C.M., Bharadwaj, R., Wong, A., Ness, K.D., Beppu, L.W., Deeg, H.J., McFarland, C., Loeb, K.R., Valente, W.J., Ericson, N.G., Stevens, E.A., Radich, J.P., Mikkelsen, T.S., Hindson, B.J., Bielas, J.H.: Massively parallel digital transcriptional profiling of single cells. Nature Communications **8**, 14049 (2017). Article
